# Diffusion tensor imaging biomarkers and clinical assessments in amyotrophic lateral sclerosis (ALS) patients: an exploratory study

**DOI:** 10.1097/MS9.0000000000002332

**Published:** 2024-07-23

**Authors:** Saharnaz Pezeshgi, Sadegh Ghaderi, Sana Mohammadi, Narges Karimi, Bentolhoda Ziaadini, Mahdi Mohammadi, Farzad Fatehi

**Affiliations:** a Neuromuscular Research Center, Department of Neurology, Shariati Hospital; b Department of Neuroscience and Addiction Studies, School of Advanced Technologies in Medicine; c Department of Medical Physics and Biomedical Engineering, School of Medicine, Tehran University of Medical Sciences, Tehran, Iran; d Department of Neurology, University Hospitals of Leicester NHS Trust, Leicester, UK

**Keywords:** amyotrophic lateral sclerosis, biomarkers, diffusion tensor imaging, motor neuron disease, neuroimaging, prognosis

## Abstract

Amyotrophic lateral sclerosis (ALS) is a neurodegenerative disease characterized by progressive loss of upper and lower motor neurons. Biomarkers are needed to improve diagnosis, gauge progression, and evaluate treatment. Diffusion tensor imaging (DTI) is a promising biomarker for detecting microstructural alterations in the white matter tracts. This study aimed to assess DTI metrics as biomarkers and to examine their relationship with clinical assessments in patients with ALS. Eleven patients with ALS and 21 healthy controls (HCs) underwent 3T MRI with DTI. DTI metrics, including fractional anisotropy (FA), mean diffusivity (MD), radial diffusivity (RD), and axial diffusivity (AD), were compared between key motor and extra-motor tract groups. Group comparisons and correlations between DTI metrics also correlated with clinical scores of disability (ALSFRS-R), muscle strength (dynamometry), and motor unit loss (MUNIX). Widespread differences were found between patients with ALS and HCs in DTI metrics, including decreased FA and increased diffusivity metrics. However, MD and RD are more sensitive metrics for detecting white matter changes in patients with ALS. Significant interhemispheric correlations between the tract DTI metrics were also observed. DTI metrics showed symmetry between the hemispheres and correlated with the clinical assessments. MD, RD, and AD increases significantly correlated with lower ALSFRS-R and MUNIX scores and weaker dynamometry results. DTI reveals microstructural damage along the motor and extra-motor regions in ALS patients. DTI metrics can serve as quantitative neuroimaging biomarkers for diagnosis, prognosis, monitoring of progression, and treatment. Combined analysis of imaging, electrodiagnostic, and functional biomarkers shows potential for characterizing disease pathophysiology and progression.

## Introduction

Highlights

- Diffusion tensor imaging (DTI) detects white matter changes in amyotrophic lateral sclerosis (ALS) with high sensitivity.
- Decreased fractional anisotropy (FA) and increased mean diffusivity (MD), radial diffusivity (RD), and axial diffusivity (AD) are linked to ALS pathology.
- MD and RD are particularly sensitive in identifying ALS-related alterations.
- DTI metrics correlate with ALS Functional Rating Scale (ALSFRS-R), motor unit number index (MUNIX), and muscle strength scores.
- The study supports DTI as a potential biomarker for ALS diagnosis and monitoring.

Amyotrophic lateral sclerosis (ALS) is a devastating neurodegenerative disorder that causes progressive loss of motor neurons, leading to death within a few years^1^. Early diagnosis and accurate monitoring of disease progression are essential to improve patient care and evaluate potential treatments^2^. However, current diagnostic criteria are based on clinical symptoms that appear after significant neuronal loss has already occurred. Therefore, there is an urgent need for biomarkers that can detect the pathology of ALS at earlier stages and track its evolution over time^3^.

One promising source of biomarkers is MRI, which can noninvasively measure structural and functional changes in the brain and spinal cord^4^. Diffusion tensor imaging (DTI) is a specialized MRI technique that quantifies the diffusion of water molecules along the white matter tracts (WMTs)^2^. DTI metrics, such as fractional anisotropy (FA), mean diffusivity (MD), radial diffusivity (RD), and axial diffusivity (AD), reflect different aspects of WM damage^5,6^. Previous DTI studies in ALS patients have shown abnormalities in some motor and non-motor regions, including the corticospinal tract (CST) and frontal lobe^7–9^. However, associations between DTI metrics and clinical measures of disease severity have been inconsistent.

Although DTI has been used to assess microstructural integrity in ALS research, advancements in other quantitative neuroimaging techniques offer even more comprehensive insights into the disease. Techniques such as MR spectroscopy (MRS) can assess metabolic changes within the brain and spinal cord, potentially revealing early signs of neurodegeneration^10^. Furthermore, functional MRI (fMRI) can map brain activity patterns and quantitative susceptibility mapping can detect changes in magnetic susceptibility^10–12^. Ongoing research uses advanced quantitative neuroimaging techniques and has proven to be beneficial for detecting minimal changes.

Another potential source of biomarkers is electrodiagnostic and electrophysiological evaluation nerve conduction study (NCS) based on electromyography (EMG), such as motor unit number index (MUNIX), compound muscle action potential (CMAP), and motor unit size index (MUSIX), which directly measure motor neuron loss and have also shown promise for tracking ALS progression^13,14^. MUNIX quantifies the number of functioning motor units^13^, CMAP measures the electrical response of muscles^15^, and MUSIX measures the size of surviving motor units^16^. Furthermore, handheld dynamometry (HHD) is another clinical biomarker for quantifying muscle strength during manual tests^17^.

A multimodal approach that combines imaging, electrodiagnostics, and clinical biomarkers can offer a more comprehensive picture of pathology and progression than any single modality alone. However, few studies have investigated the relationship between multimodal approach metrics in the same cohort of ALS patients. Hence, this study aimed to examine diffusion-based biomarkers and clinical metrics, as well as markers of motor neuron loss (MUNIX and MUSIX) and clinical disability measured by the revised ALS Functional Rating Scale (ALSFRS-R) in patients with ALS. By integrating imaging, electrodiagnostic, and clinical biomarkers in patients with ALS, we may develop a multimodal panel of sensitive indicators and biomarkers for ALS progression, which could improve the diagnosis and prognosis of patients with ALS and possibly facilitate the development and testing of new therapeutic strategies.

## Methods

### Ethics statement

This study was approved by the Ethics Committee (refer to the ethical statement). Written informed consent was obtained from all participants and all clinical investigations followed the principles outlined in the Declaration of Helsinki. This study was in line with the STROCSS criteria^18^, Supplemental Digital Content 1, http://links.lww.com/MS9/A559.

### Subjects

We enrolled 11 patients with ALS and 21 age- and sex-matched healthy controls (HCs). The patients either visited or were referred to the ALS Clinic. The patients met the definite or probable ALS diagnostic criteria according to the Awaji criteria^19^ and took routine medications during the study period. The Awaji criteria were developed to improve the accuracy of ALS diagnosis, particularly in the early stages of the disease. These criteria place greater emphasis on electrodiagnostic (EMG) findings, specifically the presence of fasciculation potentials, in conjunction with a clinical presentation suggestive of ALS.

In addition to the Awaji criteria, the inclusion criteria were designed to ensure homogeneity. The inclusion criteria were designed to ensure a homogeneous group with a confirmed ALS diagnosis, based on a combination of clinical presentation and electrodiagnostic findings. Additionally, all participants were required to be between the ages of 18 and 80. Several exclusion criteria were applied to isolate the effects of ALS on metrics of interest. Patients with significant sensory complaints suggestive of alternative neurological conditions were excluded from this study. Additionally, any participant with a co-existing history of nervous system disorders, such as multiple sclerosis or myasthenia gravis, was excluded to avoid confounding influences. Patients with a history of nerve trauma, spinal cord injury, or brain trauma were also excluded because these events can cause motor and sensory deficits that could mimic ALS symptoms. Finally, participants with any contraindications to MRI scanning, such as claustrophobia or metallic implants, were excluded to ensure safety during the imaging procedures.

### Diffusion tensor imaging

DTI of the brain was performed using a Siemens 3.0 Tesla scanner (Prisma, 2016) with a superconductive zero-helium boil-off 3T magnet at the National Brain Mapping Laboratory using a 20-channel phased-array head coil. The TRACE sequence of echo-planar imaging (EPI) was employed to generate a trace image that consolidated data from various diffusion-weighted directions. The trace image represents the mean apparent diffusion coefficient (ADC) in all directions. The diffusion-sampling scheme consisted of no-weighting (b=0 s/mm^2^) images followed by measurements along 64 non-collinear/non-coplanar directions isotropically distributed in space (b=1000 s/mm^2^ and b=2000 s/mm^2^). The other parameters were as follows: TR=9900 ms, TE=90 ms, NEX=2, FOV read=256 mm, slice=2 mm, and voxel size=2×2×2 mm^3^. The TRActs Constrained by UnderLying Anatomy (TRACULA) tool (https://surfer.nmr.mgh.harvard.edu/fswiki/Tracul), which is a component of FreeSurfer^20^, was used to process all diffusion data^21–23^. It utilizes prior knowledge about potential WM pathways gathered from manually labeled tracts in training subjects.

### Electrodiagnostic assessments

All the enrolled patients underwent electromyography and nerve conduction studies using a Nicolet Viking EDX system (Natus Neurology, Middleton). The MUNIX was measured in a 3-step process: First, the CMAP was measured by placing electrodes on the little finger and the back of the hand and stimulating the ulnar nerve from the wrist. This allowed for the measurement of the CMAP range and strength in the hypothenar, thenar, and anterior leg muscles. The surface electromyographic interference pattern (SIP) was measured in the second step while the subject performed isometric contractions at varying intensities. Software-defined networking (SDN) software was used to analyze the CMAP and SIP signals and calculate the number and size of the movement units.

### Statistics

Statistical analysis was conducted using the Student’s *t*-test or nonparametric tests for comparison. Pearson or Spearman correlation coefficients were used to assess the correlations among the groups, depending on whether the data followed a normal distribution. The significance level was set at *P* less than 0.05, and descriptive data are expressed as mean±SD. Analyses were performed using SPSS 24 (IBM Corp.) and Prism 9.0 (GraphPad Software).

## Results

Eleven patients diagnosed with ALS (four females) and 21 HCs (11 females) were included in the study. The mean age±SD of patients was 48.45±6.62 years, and in HCs was 41.86±8.82 (*P* value=0.025). The disease duration in patients with ALS was 9±9.4 months, and the mean ALSFRS-R was 39.4±5.7. The clinical parameters of patients with ALS and HCs are presented in Table 1.

**Table 1:** Clinical measures of amyotrophic lateral sclerosis patients

Descriptive statistics			
	Minimum	Maximum	Mean±SD
MUNIXLmedian	9	155	91±74.9
MUNIXLulnar	16	176	101.6±57.4
MUNIXLtibal	23	111	80.3±32.5
MUSIXRtibal	33	184	65.2±45.4
MUSIXRulnar	36	246	101±64.1
MUSIXRmedian	40	410	131.3±110.2
MUSIXLmedian	42	75	58±15.6
MUSIXLulnar	42	141	81.9±40.5
MUSIXLtibial	37	61	49.6±6.9
ALSFRS-R	27	46	39.4±5.6
DynamoR	1	36	18.4±11.9
DynamoL	10	40	23.3±9.9
Duration (month)	2	36	9

ALS Functional Rating Scale; MUSIX, motor unit size index.

We computed the correlation coefficient between DTI metrics of brain regions as well as between clinical parameters and DTI metrics of specific regions. The study results were based on the most commonly occurring areas that exhibited a significant correlation coefficient with other brain regions and clinical parameters, and these findings have been thoroughly discussed. Regions with less frequent correlations are detailed in Supplements 2, Supplemental Digital Content 2, http://links.lww.com/MS9/A560 and 3, Supplemental Digital Content 3, http://links.lww.com/MS9/A561, respectively.

### Comparison of DTI metrics

**Figure 1:**
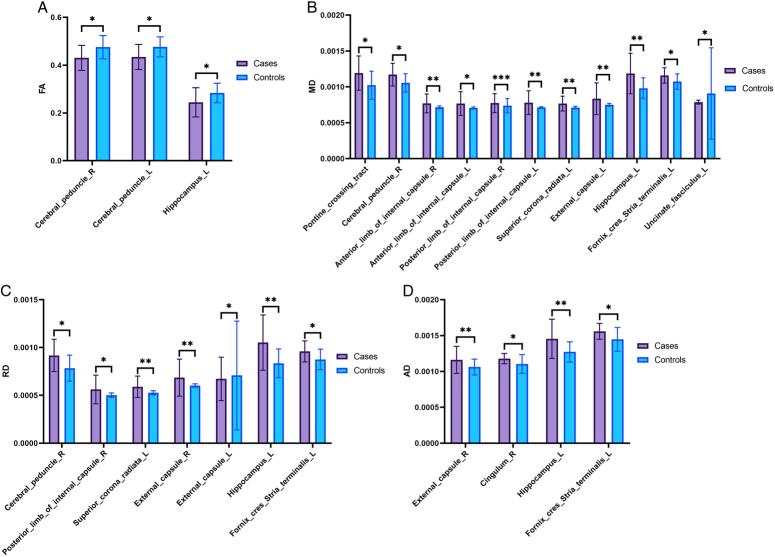
Most of the significant differences in (A) FA, (B) MD, (C) RD, and (D) AD metrics (A *P* value of less than 0.05 is shown with a single star (*). A *P* value less than 0.01, denoted by two stars (**). A *P* value of less than 0.001 is denoted by three stars (***)). AD, axial diffusivity; FA, fractional anisotropy; MD, mean diffusivity; RD, radial diffusivity.

The comparison of biomarkers/metrics FA, MD, RD, and AD between the patient and control groups revealed that the MD metric showed a significant difference in more regions than did the other metrics (Table 2). FA decreased significantly in some regions, such as the bilateral cerebral peduncle and left hippocampus. Simultaneously, MD increased significantly in various regions, including the pontine crossing tract, right cerebral peduncle, bilateral anterior and posterior limbs of the internal capsule, left external capsule, left superior corona radiata, left hippocampus, and left fornix cres stria terminalis. RD increased significantly in various regions, including the right cerebral peduncle, right posterior limb of the internal capsule, bilateral external capsule, left superior corona radiata, left hippocampus, and left fornix cres stria terminalis. AD was also significantly increased in various regions, including the right external capsule, left hippocampus, left fornix, cres stria terminalis, and right cingulum. However, the left uncinate fasciculus and right external capsule in MD, the left posterior limb of the internal capsule in RD, and the left posterior corona radiata in AD showed a decrease. The significant results are shown in Figure 1, and a comparison of imaging biomarkers from all regions can be found in Supplementary Tables S1–S4 (Supplement 1, Supplemental Digital Content 4, http://links.lww.com/MS9/A562).

**Table 2:** Comparison of DTI metrics between amyotrophic lateral sclerosis and healthy controls groups

Imaging parameters	Cases (ALS)		Controls (HCs)		*P*
	Mean	SD	Mean	SD	
FA					
Cerebral_peduncle_R	0.4302	0.0522	0.4752	0.0484	0.021*
Cerebral_peduncle_L	0.4341	0.0524	0.4765	0.0418	0.018*
Hippocampus_L	0.2443	0.0613	0.2836	0.0409	0.041*
MD					
Pontine_crossing_tract	0.0012	0.0002	0.0010	0.0002	0.042*
Cerebral_peduncle_R	0.0012	0.0002	0.0011	0.0001	0.045*
Anterior_limb_of_internal_capsule_R	0.0008	0.0001	0.0007	0.0000	0.009**
Anterior_limb_of_internal_capsule_L	0.0008	0.0002	0.0007	0.0000	0.015*
Posterior_limb_of_internal_capsule_R	0.0008	0.0001	0.0007	0.0001	0.001***
Posterior_limb_of_internal_capsule_L	0.0008	0.0002	0.0007	0.0000	0.003**
Superior_corona_radiata_L	0.0008	0.0001	0.0007	0.0000	0.004**
External_capsule_R	0.0008	0.0002	0.0139	0.0601	0.004**
External_capsule_L	0.0008	0.0002	0.0008	0.0000	0.009**
Hippocampus_L	0.0012	0.0003	0.0010	0.0001	0.003**
Fornix_cres_Stria_terminalis_L	0.0012	0.0001	0.0011	0.0001	0.030*
Uncinate_fasciculus_L	0.0008	0.0000	0.0009	0.0006	0.037*
RD					
Cerebral_peduncle_R	0.0009	0.0002	0.0008	0.0001	0.024*
Posterior_limb_of_internal_capsule_R	0.0006	0.0001	0.0005	0.0000	0.028*
Posterior_limb_of_internal_capsule_L	0.0006	0.0002	0.0295	0.1330	0.012*
Superior_corona_radiata_L	0.0006	0.0001	0.0005	0.0000	0.008**
External_capsule_R	0.0007	0.0002	0.0006	0.0000	0.002**
External_capsule_L	0.0007	0.0002	0.0007	0.0006	0.023*
Hippocampus_L	0.0011	0.0003	0.0008	0.0002	0.003*
Fornix_cres_Stria_terminalis_L	0.0010	0.0001	0.0009	0.0001	0.048*
AD					
Posterior_corona_radiata_L	0.0013	0.0000	0.0083	0.0314	0.010**
External_capsule_R	0.0012	0.0002	0.0011	0.0001	0.010**
Cingulum_R	0.0012	0.0001	0.0011	0.0001	0.037*
Hippocampus_L	0.0015	0.0003	0.0013	0.0001	0.009**
Fornix_cres_Stria_terminalis_L	0.0016	0.0001	0.0014	0.0002	0.048*

A *P* value that is less than 0.05 is indicated by a single star (*).

A *P* value that is less than 0.01 is represented by two stars (**).

A *P* value that is less than 0.001 is represented by three stars (***).

AD, axial diffusivity; ALS, amyotrophic lateral sclerosis; DTI, diffusion tensor imaging; FA, fractional anisotropy; HC, healthy control; MD, mean diffusivity; RD, radial diffusivity.

### Correlations of DTI metrics

**Figure 2:**
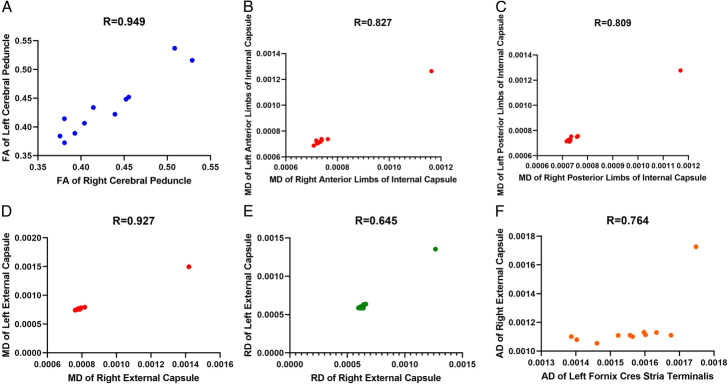
Some significant correlations of diffusion tensor imaging metrics between tracts (the alteration in these tracts between the two groups is significant; refer to Table 3). AD, axial diffusivity; FA, fractional anisotropy; MD, mean diffusivity; RD, radial diffusivity.

When examining the correlations between imaging metrics, we observed a significant association between the DTI biomarkers in multiple pairs of regions. Figure 2 shows some significant correlations in the imaging metrics between pairs of regions, specifically bilateral regions. Supplementary Tables S5–S8 (Supplement 2, Supplemental Digital Content 2, http://links.lww.com/MS9/A560) comprehensively list all significant correlations.

### Correlations between DTI metrics and clinical parameters

**Figure 3:**
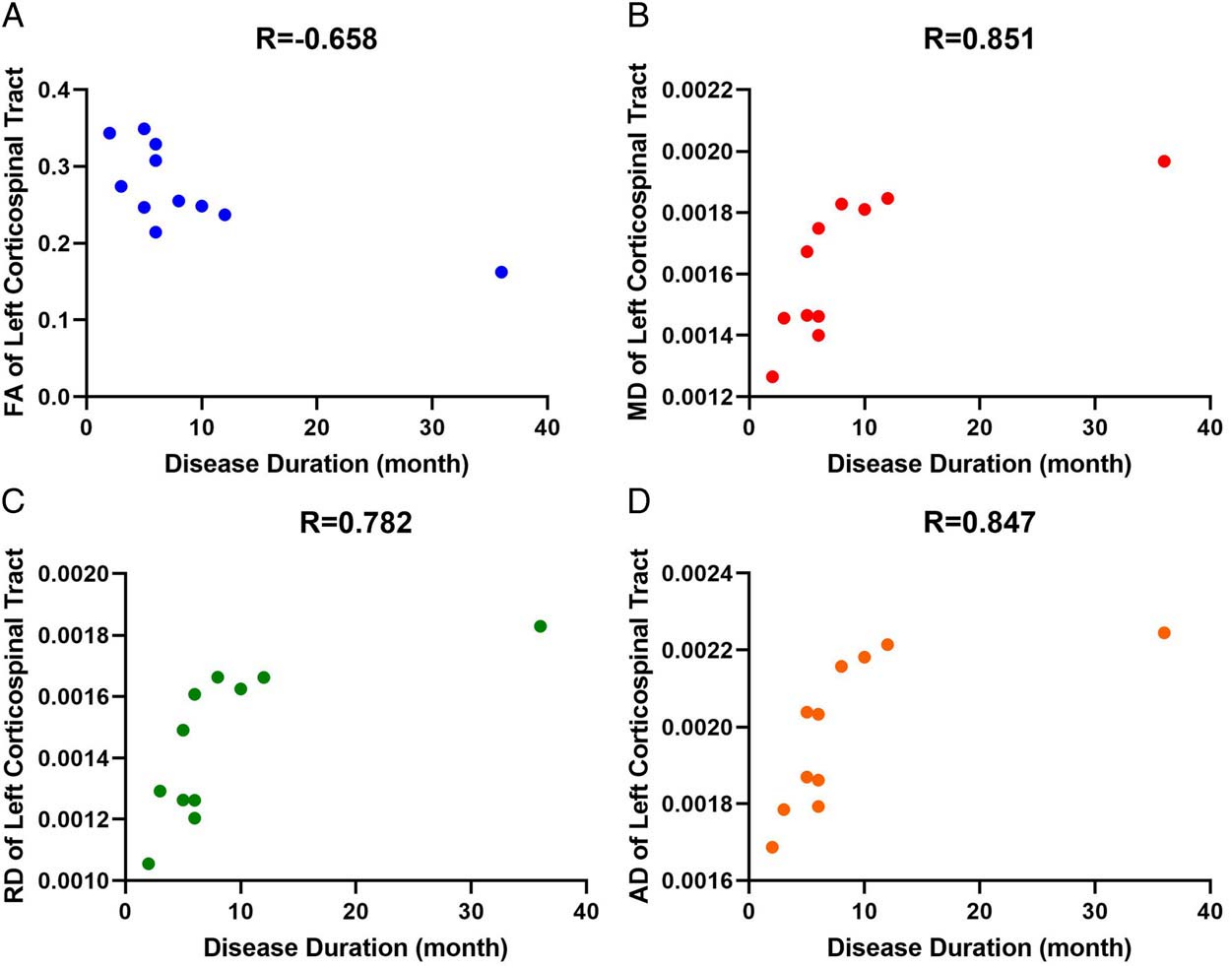
Significant correlations between disease duration and diffusion tensor imaging metrics of left corticospinal tract. AD, axial diffusivity; FA, fractional anisotropy; MD, mean diffusivity; RD, radial diffusivity.

**Figure 4:**
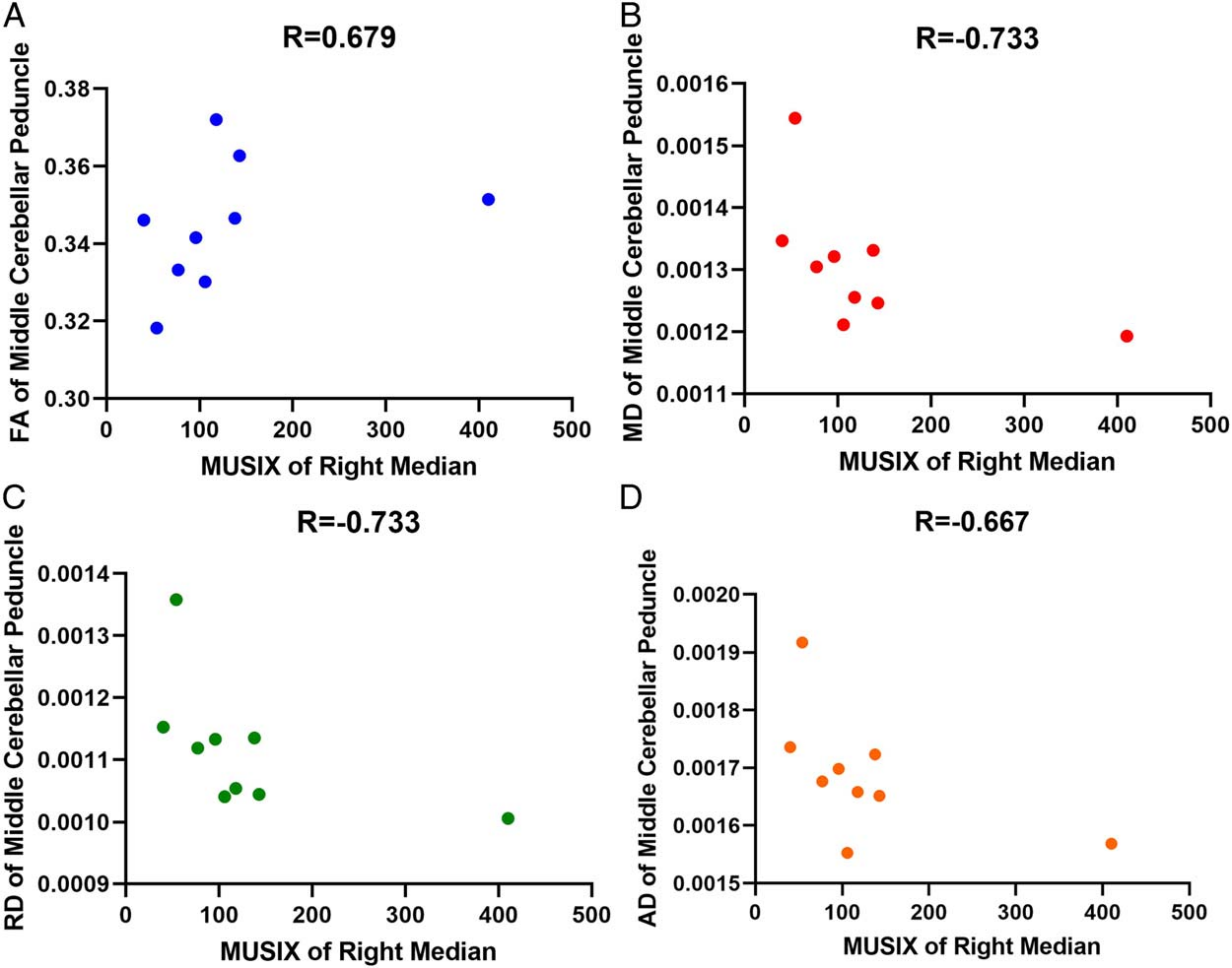
Significant correlations between the right MUSIX median and diffusion tensor imaging metrics of the middle cerebellar peduncle. AD, axial diffusivity; FA, fractional anisotropy; MD, mean diffusivity; MUSIX, motor unit size index; RD, radial diffusivity.

**Figure 5:**
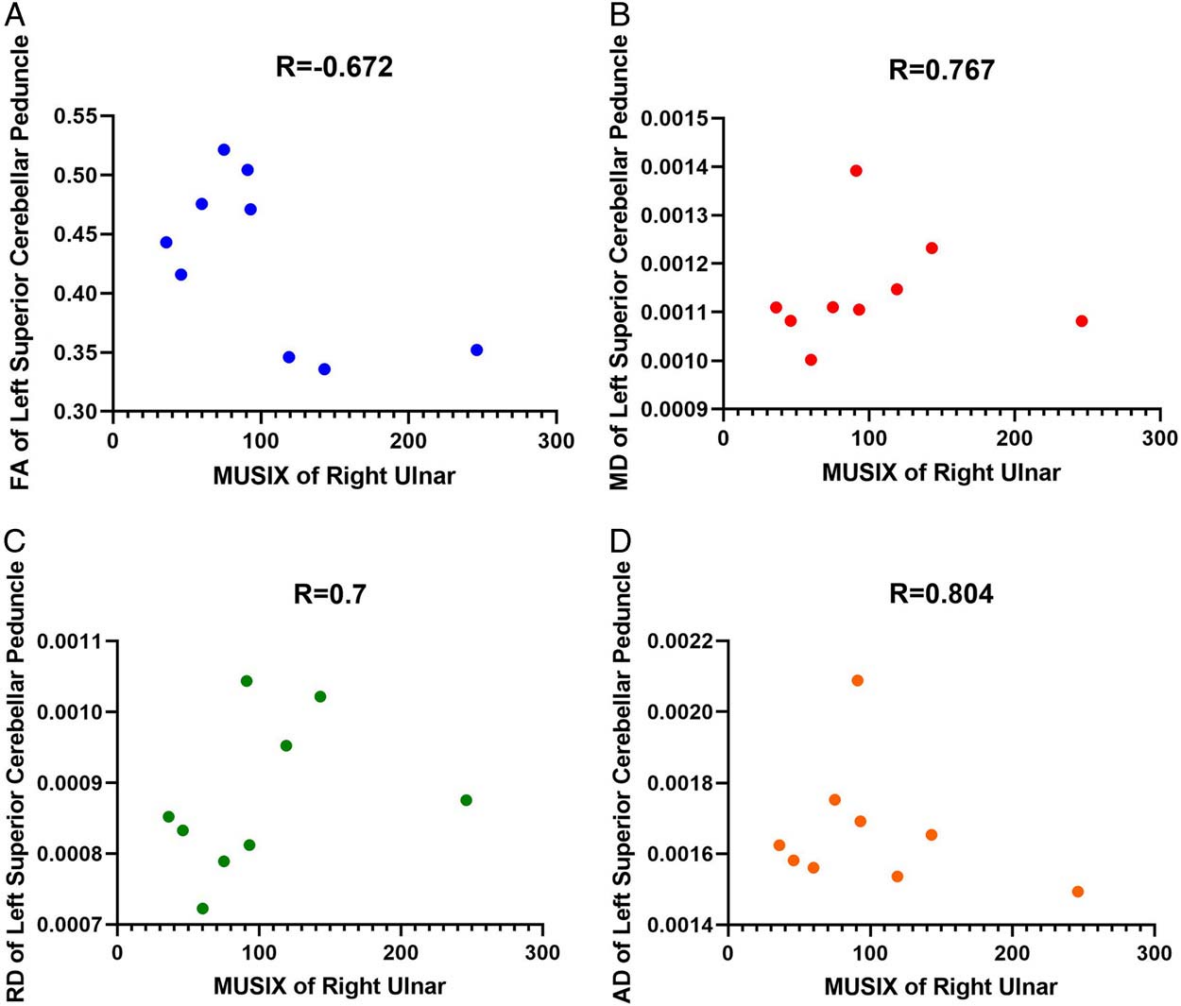
Significant correlations between right MUSIX ulnar and left diffusion tensor imaging metrics of superior cerebellar peduncle. AD, axial diffusivity; FA, fractional anisotropy; MD, mean diffusivity; MUSIX, motor unit size index; RD, radial diffusivity.

The clinical and DTI metrics exhibited significant connections in multiple regions. Figures 3–5 provide examples of significant correlations between the imaging data and clinical parameters for each metric. Supplementary Tables S9–S12 (Supplement 3, Supplemental Digital Content 3, http://links.lww.com/MS9/A561) demonstrate all significant relationships between the clinical and DTI metrics.

### Synthesis of findings

Significant alterations in DTI diagnostic biomarkers were observed bilaterally in important brain regions of patients with ALS. These changes included a decrease in FA in the cerebral peduncle, an increase in MD in the anterior and posterior limb areas of the internal capsule, changes in MD of the external capsule, and an increase in RD in the external capsule. In addition to the significant differences between the patient and healthy groups, there was a significant correlation between the right and left hemispheres in these tracts of the patient’s brain. Table 3 lists all tracts that showed significant differences between the two groups and the correlation between these tracts using DTI biomarkers.

**Table 3:** Tracts with significant alterations between groups and their correlation in patient brains based on DTI biomarkers

Metrics	Tracts (regions)	(1) Patients control (mean±SD) *P*	(2) Patients control (mean±SD) *P*	Correlation (1) and (2) in patients (R), *P*
FA^a^	(1) Right cerebral peduncle (2) Left cerebral peduncle	0.4302±0.0522 0.4752±0.0484 (0.021*)	0.4341±0.0524 0.4765±0.0418 (0.018*)	R=0.949, *P*=0.000
MD^a^	(1) Right anterior limb of internal capsule (2) Left anterior limb of internal capsule	0.0008±0.0001 0.0007±0.0000 (0.009**)	0.0008±0.0002 0.0007±0.0000 (0.015*)	R=0.827, *P*=0.020
MD	(1) Right anterior limb of internal capsule (2) Right posterior limb of internal capsule	0.0008±0.0001 0.0007±0.0000 (0.009**)	0.0008±0.0001 0.0007±0.0001 (0.001***)	R=0.764, *P*=0.006
MD	(1) Right anterior limb of internal capsule (2) Left posterior limb of internal capsule	0.0008±0.0001 0.0007±0.0000 (0.009**)	0.0008±0.0002 0.0007±0.0000 (0.003**)	R=0.718, *P*=0.013
MD	(1) Right anterior limb of internal capsule (2) Left superior corona radiata	0.0008±0.0001 0.0007±0.0000 (0.009**)	0.0008±0.0002 0.0007±0.0000 (0.015*)	R=0.864, *P*=0.001
MD	(1) Right anterior limb of internal capsule (2) Right external capsule	0.0008±0.0001 0.0007±0.0000 (0.009**)	0.0008±0.0002 0.0139±0.0601 (0.004**)	R=0.791, *P*=0.004
MD	(1) Right anterior limb of internal capsule (2) Left external capsule	0.0008±0.0001 0.0007±0.0000 (0.009**)	0.0008±0.0002 0.0008±0.0000 (0.009**)	R=0.791, *P*=0.004
MD	(1) Left anterior limb of internal capsule (2) Left posterior limb of internal capsule	0.0008±0.0002 0.0007±0.0000 (0.015*)	0.0008±0.0002 0.0007±0.0000 (0.015*)	R=0.618, *P*=0.043
MD	(1) Left anterior limb of internal capsule (2) Left superior corona radiata	0.0008±0.0002 0.0007±0.0000 (0.015*)	0.0008±0.0002 0.0007±0.0000 (0.015*)	R=0.736, *P*=0.010
MD^a^	(1) Right posterior limb of internal capsule (2) Left posterior limb of internal capsule	0.0008±0.0001 0.0007±0.0001 (0.001***)	0.0008±0.0002 0.0007±0.0000 (0.003**)	R=0.809, *P*=0.003
MD	(1) Left superior corona radiata (2) Right external capsule	0.0008±0.0002 0.0007±0.0000 (0.015*)	0.0008±0.0002 0.0139±0.0601 (0.004**)	R=0.645, *P*=0.032
MD	(1) Left superior corona radiata (2) Left external capsule	0.0008±0.0002 0.0007±0.0000 (0.015*)	0.0008±0.0002 0.0008±0.0000 (0.009**)	R=0.782, *P*=0.004
MD^a^	(1) Right external capsule (2) Left external capsule	0.0008±0.0002 0.0139±0.0601 (0.004**)	0.0008±0.0002 0.0008±0.0000 (0.009**)	R=0.927, *P*=0.000
MD	(1) Left fornix cres stria terminalis (2) Left superior corona radiata	0.0012±0.0001 0.0011±0.0001 (0.030*)	0.0008±0.0002 0.0007±0.0000 (0.015*)	R=0.618, *P*=0.043
MD	(1) Left uncinate fasciculus (2) Right external capsule	0.0008±0.0000 0.0009±0.0006 (0.037*)	0.0008±0.0002 0.0139±0.0601 (0.004**)	R=0.655, *P*=0.029
RD	(1) Left superior corona radiata (2) Left external capsule	0.0006±0.0001 0.0005±0.0000 (0.008**)	0.0007±0.0002 0.0007±0.0006 (0.023*)	R=0.727, *P*=0.011
RD^a^	(1) Right external capsule (2) Left external capsule	0.0007±0.0002 0.0006±0.0000 (0.002**)	0.0007±0.0002 0.0007±0.0006 (0.023*)	R=0.645, *P*=0.032
RD	(1) Left fornix cres stria terminalis (2) Left superior corona radiata	0.0010±0.0001 0.0009±0.0001 (0.048*)	0.0006±0.0001 0.0005±0.0000 (0.008**)	R=0.627, *P*=0.039
AD	(1) Left fornix cres stria terminalis (2) Right external capsule	0.0016±0.0001 0.0014±0.0002 (0.048*)	0.0012±0.0002 0.0011±0.0001 (0.010**)	R=0.764, *P*=0.006

A *P* value that is less than 0.05 is indicated by a single star (*).

A *P* value that is less than 0.01 is represented by two stars (**).

A *P* value that is less than 0.001 is represented by three stars (***).

AD, axial diffusivity; DTI, diffusion tensor imaging; FA, fractional anisotropy; MD, mean diffusivity; RD, radial diffusivity.

a Bilateral.

A positive correlation existed between MD increases in the left hippocampus and left dynamo (0.714). In contrast, the pontine crossing tract had a negative correlation with the right dynamo (−0.667). Additionally, there was a negative correlation between MD increase in the left posterior limb of the internal capsule and ALSFRS-R (−0.696). The same negative correlation was observed between the right posterior limb of the internal capsule and ALSFRS-R (−0.769). In addition, increases in RD in the left hippocampus were positively correlated with left MUNIX tibial (0.762).

Similarly, the right external capsule and left superior corona radiata were positively correlated with the left MUNIX tibial (0.762) and left MUNIX ulnar (0.786). Lastly, RD decreased in the left posterior limb of the internal capsule, having a negative correlation with the left MUSIX tibial (−0.731), whereas AD increased in the right cingulum and negatively correlated with the right MUSIX ulnar (−0.706). Table 4 shows the significant changes in tract-imaging biomarkers correlated with clinical parameters.

**Table 4:** Significant tract diffusion tensor imaging biomarker alterations and correlation with clinical parameters

Tract	Biomarkers alteration patients/controls	Correlation with clinical parameters (R)
Left hippocampus	MD ↑	Dynamo-Left (0.714)
	RD ↑	MUNIX-Left-tibial (0.762)
Left posterior limb of internal capsule	MD ↑	ALSFRS-R (−0.696)
	RD ↓	MUSIX-Left-tibial (−0.731)
Right posterior limb of internal capsule	MD ↑	ALSFRS-R (−0.769)
Pontine crossing tract	MD ↑	Dynamo-Right (−0.667)
Right external capsule	RD ↑	MUNIX-Left-tibial (0.762)
Left superior corona radiata	RD ↑	MUNIX-Left-ulnar (0.786)
Right cingulum	AD ↑	MUSIX-Right-ulnar (−0.706)

AD, axial diffusivity; ALSFRS-R, ALS Functional Rating Scale; MD, mean diffusivity; MUNIX, motor unit number index; MUSIX, motor unit size index; RD, radial diffusivity.

## Discussion

DTI revealed significant alterations in key WMTs of patients with ALS. These alterations manifest as decreased FA and diffusivity metrics, including increased MD, RD, and AD. These findings suggest that DTI metrics hold promise as biomarkers for assessing disease progression and prognosis in both the cortical and subcortical brain regions. Furthermore, our study underscores the importance of a multimodal approach that integrates imaging, electrodiagnostics, and clinical biomarkers. This approach has yielded a more comprehensive understanding of the pathology and progression of ALS. Notably, the combination of DTI metrics with clinical measures of disease severity yielded an improved prediction of clinical prognosis in patients with ALS. These findings suggest that a multimodal approach may be a valuable tool for monitoring disease progression and potentially informing the development of personalized treatment strategies for ALS patients.

Our study may contribute to the growing body of literature on ALS biomarkers by highlighting the potential of DTI as a non-invasive technique that can be used to detect microstructural abnormalities in the brain^5,24–29^. These microstructural abnormalities are thought to be due to progressive degeneration of motor neurons in ALS^30^. As motor neurons degenerate, they lose their ability to maintain myelin sheaths around their axons^28^. This phenomenon leads to decreased axonal conduction velocity and increased diffusion of water molecules, which can be detected using DTI^28,31^. These abnormalities are present in ALS patients even in the early stages of the disease and can be used to monitor disease progression, such as tracking the rate of decline in axonal integrity over time^32^. Our study aligns with previous findings and provides valuable information that can be used to identify biomarkers that predict treatment response.

The regions most affected in ALS patients were the pontine crossing tract, cerebral peduncle, anterior and posterior limbs of the internal capsule, external capsule, superior corona radiata, left hippocampus, and fornix cress stria terminalis. Impairment of this region causes motor and cognitive dysfunction, which are affected in ALS^2,33,34^.

The most notable finding was the widespread increase in MD and RD in ALS patients compared to HCs, seen in critical motor and extra-motor regions such as the left external capsule, right posterior limb of the internal capsule, corona radiata, right cerebral peduncle, left fornix cress stria terminalis, and left hippocampal tract. This finding aligns with prior research showing increased biomarkers reflecting greater overall diffusion in ALS, likely indicating axonal loss, degeneration, and myelin breakdown^5,35^, suggesting increased water diffusivity and potentially disrupted microstructural organization.

We also found that some of the DTI metrics we studied correlated with clinical parameters in patients with ALS. For example, increases in MD are correlated with muscle strength on dynamometry, supporting the use of MD as a biomarker of lower motor dysfunction^36^. Clinically, MD increases in the left hippocampus were positively correlated with the left dynamometer, whereas RD increases in the left hippocampus were positively correlated with the left MUNIX tibial. These findings suggest that DTI metrics may be used to predict clinical outcomes in patients with^5,37^.

Meanwhile, an increase in MD in regions such as the posterior limb of the internal capsule correlated negatively with functional scales such as the ALSFRS-R. Furthermore, increased RD in areas such as the right external capsule and left superior corona radiata correlated positively with the left tibial and ulnar MUNIX. These findings suggest that changes in WM integrity captured by DTI biomarkers may be linked to the clinical manifestations and disease progression in ALS^8,28,38,39^. Understanding these relationships may assist in predicting disease prognosis and monitoring treatment response.

As noted, MD and RD provided more evidence than FA and AD did. FA only decreased in limited regions, such as the bilateral cerebral peduncle and left hippocampal tract, confirming that MD and RD may detect WM changes in ALS earlier than FA. The increased MD and RD patterns across motor and extra-motor networks provide insights into the anatomical distribution of ALS pathology^5,28,29,40^. An increase in AD was observed in several regions, including the left hippocampus, right external capsule, left fornix cress stria terminalis, and right cingulum. Additionally, an increase in AD in the right cingulum negatively correlated with the right MUSIX ulnar. However, a decrease was observed in the left uncinate fasciculus and right external capsule in MD, the left posterior limb of the internal capsule in RD, and the left posterior corona radiata in AD. These widespread changes in diffusion metrics in WMTs throughout the brain reflect diffuse axonal injury and degeneration involved in ALS pathogenesis. Both increases and decreases were observed, indicating complex dynamics between degenerative and compensatory changes across the WM^24,41^.

We discovered associations between DTI biomarkers and parameters such as the ALSFRS-R and MUNIX scores. The negative correlation of MD and RD with increasing ALSFRS-R score is clinically significant, indicating the prognostic value of these metrics in predicting disease severity and progression rate. Increased RD in motor tracts is also positively correlated with MUNIX amplitudes, potentially indicating compensatory mechanisms as motor neurons decline. Overall, these correlations highlight the clinical utility of DTI in assessing ALS^42,43^.

The correlation between the duration of the disease and the left CST suggests that, as the disease lasts longer, there is a decrease in FA (and an increase in other metrics) in the left CST, indicating more severe damage or loss of axons in this pathway. This finding aligns with previous research showing lower FA in the CST of ALS patients than in healthy individuals and a negative correlation between FA and disease duration^7,9,44^. Furthermore, the positive correlation between the right MUSIX ulnar and the left superior cerebellar peduncle suggests that higher MUSIX ulnar is associated with higher MD, RD, and AD in the left superior cerebellar peduncle, indicating more severe damage or inflammation in this region (Fig. 4). More significant motor unit loss and increased motor unit size are linked to increases in MUSIX, but muscle strength is relatively preserved, indicating that MUSIX can be used as a quantitative measure of re-innervation in clinical trials. Moreover, MUSIX suggests that re-innervation may influence the progression of weakness may be influenced by re-innervation^13,45^. However, the negative correlation between the right MUSIX median and the middle cerebellar peduncle suggests that a lower MUSIX median is associated with higher MD, RD, and AD in the middle cerebellar peduncle, indicating potential ulnar nerve regarding that median nerve was more affected in ALS patients, especially right side (ulnar˃tibial˃median) (Supplement 3, Supplemental Digital Content 3, http://links.lww.com/MS9/A561 and Figs. 4 and 5). Future multimodal and multiparametric longitudinal studies should be conducted to validate these findings.

Another key finding was the correlation between the contralateral regions in patients with ALS, with symmetric alterations in metrics between the right and left hemispheres. This bilateral symmetry of diffusion changes likely reflects the relatively symmetric spread of upper and lower motor neuron degeneration, which pathologically characterizes ALS. Additionally, these findings suggest that ALS is a bilateral disorder that affects both hemispheres of the brain, consistent with previous studies that reported bilateral involvement of brain regions in ALS^5,27^. Tracking this symmetric involvement through DTI could help gauge the overall disease progression. However, some studies have reported asymmetrical degeneration patterns in ALS. Therefore, further research is needed to elucidate the factors that influence the symmetry or asymmetry of degeneration in ALS.

While this study provides evidence that DTI metrics may serve as useful biomarkers for ALS, some limitations need to be addressed in future research. The cross-sectional design and relatively small sample size limited the generalizability and causal inference of our findings. Longitudinal studies with larger, more diverse cohorts are needed to establish the temporal relationships between DTI metrics and disease progression. Multimodal neuroimaging approaches combining DTI with other advanced MRI techniques as well as electrophysiological, genetic, and clinical measures should be explored for a more comprehensive characterization of ALS pathogenesis and prognosis. The practical clinical application of imaging biomarkers faces the challenges of accessibility and validation that need to be overcome. Overall, this study provides a foundation for further research to develop optimized prognostic models incorporating combinations of neuroimaging, electrodiagnostics, and clinical metrics tailored to individual patient contexts. Larger multiparametric efforts are warranted to validate DTI and other advanced MRI techniques as clinically useful biomarkers of ALS.

## Conclusions

This study highlights the potential of DTI as a sensitive neuroimaging biomarker of ALS. These findings indicate widespread microstructural WM alterations in motor and extra-motor regions in patients with ALS compared to HCs, reflecting underlying axonal degeneration, myelin breakdown, and loss of fiber integrity. The involvement of both hemispheres suggests a bilateral nature of the disease. Furthermore, DTI metrics showed significant correlations with clinical assessments, such as ALSFRS-R, MUNIX, and dynamometry, indicating their value in tracking progression and disease severity. Combining neuroimaging, electrophysiological, and clinical evaluations can provide a more comprehensive understanding of the heterogeneity of ALS pathology in each patient.

## Ethical approval

Ethics Committee of Tehran University of Medical Sciences approved human studies (Ethical Code: IR.TUMS.MEDICINE.REC.1399.930), and participants provided written informed consent for publication.

## Consent

Written informed consent was obtained from the patient for publication of this case report and accompanying images. A copy of the written consent is available for review by the Editor-in-Chief of this journal on request.

## Source of funding

The authors did not receive any specific funding for this manuscript.

## Author contribution

S.P.: conceptualization, methodology / study design, data curation, writing—original draft preparation, visualization, investigation, supervision, validation, writing—reviewing, and editing. S.G.: conceptualization, methodology / study design, data curation, writing—original draft preparation, visualization, investigation, supervision, validation, writing—reviewing, and editing. S.M.: conceptualization, methodology / study design, data curation, formal analysis, software, writing—original draft preparation, visualization, investigation, writing—reviewing, and editing. N.K.: investigation and data curation. B.Z.: investigation and data curation. M.M.: formal analysis, software, and writing—original draft. F.F.: conceptualization, methodology, supervision, validation, writing—reviewing, and editing.

## Conflicts of interest disclosure

The authors declares no conflicts of interest.

## Guarantor

Prof. Farzad Fatehi.

## Data availability statement

This article contains all of the data produced or analyzed during this investigation. Any further inquiries should be forwarded to the corresponding author.

## Supplementary Material

## References

[1] Ghaderi S Batouli SAH Mohammadi S. Iron quantification in basal ganglia using quantitative susceptibility mapping in a patient with ALS: a case report and literature review. Front Neurosci 2023;17:1229082. 37877011 10.3389/fnins.2023.1229082 PMC10593460

[2] Ghaderi S Fatehi F Kalra S. MRI biomarkers for memory-related impairment in amyotrophic lateral sclerosis: a systematic review. Amyotroph Later Scler Frontotemp Degener 2023;24:572–588. 10.1080/21678421.2023.2236651 37469125

[3] Mohammadi S Ghaderi S. Motor band sign in motor neuron diseases using magnetic resonance imaging: a systematic review. Acta Neurol Scand 2023;2023:e6677967.

[4] Mohammadi S Ghaderi S. Parkinson’s disease and Parkinsonism syndromes: evaluating iron deposition in the putamen using magnetic susceptibility MRI techniques—a systematic review and literature analysis. Heliyon 2024;10:e27950. 38689949 10.1016/j.heliyon.2024.e27950 PMC11059419

[5] Baek S-H Park J Kim YH. Usefulness of diffusion tensor imaging findings as biomarkers for amyotrophic lateral sclerosis. Sci Rep 2020;10:5199. 32251314 10.1038/s41598-020-62049-0 PMC7090054

[6] Silvagni E Inglese F Bortoluzzi A. Longitudinal changes in cerebral white matter microstructure in newly diagnosed systemic lupus erythematosus patients. Rheumatology (Oxford) 2020;60:2678–2687. 10.1093/rheumatology/keaa677 PMC8213425 33507240

[7] Agosta F Pagani E Petrolini M. Assessment of white matter tract damage in patients with amyotrophic lateral sclerosis: a diffusion tensor MR imaging tractography study. AJNR Am J Neuroradiol 2010;31:1457–1461. 20395382 10.3174/ajnr.A2105 PMC7966122

[8] Alruwaili AR Pannek K Henderson RD. Tract integrity in amyotrophic lateral sclerosis: 6–month evaluation using MR diffusion tensor imaging. BMC Med Imaging 2019;19:19. 30795741 10.1186/s12880-019-0319-3 PMC6387547

[9] Rajagopalan V Pioro EP. Differential involvement of corticospinal tract (CST) fibers in UMN-predominant ALS patients with or without CST hyperintensity: a diffusion tensor tractography study. NeuroImage: Clin 2017;14:574–579. 28337412 10.1016/j.nicl.2017.02.017 PMC5349615

[10] Mohammadi S Ghaderi S Fatehi F. MRI biomarkers and neuropsychological assessments of hippocampal and parahippocampal regions affected by ALS: a systematic review. CNS Neurosci Ther 2024;30:e14578. 38334254 10.1111/cns.14578 PMC10853901

[11] Mohammadi S Ghaderi S Fatehi F. Quantitative susceptibility mapping as an early neuroimaging biomarker for amyotrophic lateral sclerosis: a review. iRADIOLOGY early view 2024. 10.1002/ird3.88

[12] Mohammadi S Ghaderi S Fatehi F. Putamen iron quantification in diseases with neurodegeneration: a meta-analysis of the quantitative susceptibility mapping technique. Brain Imaging Behav 2024. 10.1007/s11682-024-00895-6 38758278

[13] Chan Y Alix JJP Neuwirth C. Reinnervation as measured by the motor unit size index is associated with preservation of muscle strength in amyotrophic lateral sclerosis, but not all muscles reinnervate. Muscle Nerve 2022;65:203–210. 34687220 10.1002/mus.27444

[14] Okhovat AA Advani S Moradi K. Application of CMAP scan for the evaluation of patients with chronic inflammatory demyelinating polyneuropathy: a prospective study. Neurophysiol Clin 2021;51:175–181. 33423829 10.1016/j.neucli.2020.12.005

[15] Pollari E Prior R Robberecht W. In vivo electrophysiological measurement of compound muscle action potential from the forelimbs in mouse models of motor neuron degeneration. J Vis Exp 2018;136:57741. 10.3791/57741 PMC6101751 29985328

[16] Uslu S Nüzket T Uysal H. Modified motor unit number index (MUNIX) algorithm for assessing excitability of alpha motor neuron in spasticity. Clin Neurophysiol Pract 2018;3:127–133. 30215023 10.1016/j.cnp.2018.05.002 PMC6134175

[17] Shefner JM Liu D Leitner ML. Quantitative strength testing in ALS clinical trials. Neurology 2016;87:617–624. 27385750 10.1212/WNL.0000000000002941 PMC4977369

[18] Mathew G Agha R Albrecht J STROCSS Group. STROCSS 2021: Strengthening the reporting of cohort, cross-sectional and case-control studies in surgery. Int J Surg 2021;96:106165. 34774726 10.1016/j.ijsu.2021.106165

[19] Costa J Swash M de Carvalho M. Awaji criteria for the diagnosis of amyotrophic lateral sclerosis: a systematic review. Arch Neurol 2012;69:1410–1416. 22892641 10.1001/archneurol.2012.254

[20] Fischl B. FreeSurfer. Neuroimage 2012;62:774–781. 22248573 10.1016/j.neuroimage.2012.01.021 PMC3685476

[21] Maffei C Lee C Planich M. Using diffusion MRI data acquired with ultra-high gradient strength to improve tractography in routine-quality data. Neuroimage 2021;245:118706. 34780916 10.1016/j.neuroimage.2021.118706 PMC8835483

[22] Yendiki A Panneck P Srinivasan P. Automated probabilistic reconstruction of white-matter pathways in health and disease using an atlas of the underlying anatomy. Front Neuroinform 2011;5:23. 22016733 10.3389/fninf.2011.00023 PMC3193073

[23] Andica C Kamagata K Aoki S. Automated three-dimensional major white matter bundle segmentation using diffusion magnetic resonance imaging. Anat Sci Int 2023;98:318–336. 37017902 10.1007/s12565-023-00715-9 PMC10256641

[24] Mazón M Vázquez Costa JF Ten-Esteve A. Imaging biomarkers for the diagnosis and prognosis of neurodegenerative diseases. the example of amyotrophic lateral sclerosis. Front Neurosci 2018;12:784. 30410433 10.3389/fnins.2018.00784 PMC6209630

[25] El Mendili MM Grapperon A-M Dintrich R. Alterations of microstructure and sodium homeostasis in fast amyotrophic lateral sclerosis progressors: a brain DTI and sodium MRI study. Am J Neuroradiol 2022;43:984–990. 35772800 10.3174/ajnr.A7559 PMC9262065

[26] Reyes-Leiva D Dols-Icardo O Sirisi S. Pathophysiological underpinnings of extra-motor neurodegeneration in amyotrophic lateral sclerosis: new insights from biomarker studies. Front Neurol 2022;12:750543. 35115992 10.3389/fneur.2021.750543 PMC8804092

[27] Behler A Lulé D Ludolph AC. Longitudinal monitoring of amyotrophic lateral sclerosis by diffusion tensor imaging: power calculations for group studies. Front Neurosci 2022;16:929151. 36117627 10.3389/fnins.2022.929151 PMC9479493

[28] Behler A Müller H-P Ludolph AC. Diffusion tensor imaging in amyotrophic lateral sclerosis: machine learning for biomarker development. Int J Mol Sci 2023;24:1911. 36768231 10.3390/ijms24031911 PMC9915541

[29] Hsueh S Chao C Chen T. Brain imaging signatures in amyotrophic lateral sclerosis: correlation with peripheral motor degeneration. Ann Clin Transl Neurol 2023;10:1456–1466. 37340732 10.1002/acn3.51835 PMC10424648

[30] Trojsi F Caiazzo G Corbo D. Microstructural changes across different clinical milestones of disease in amyotrophic lateral sclerosis. PLoS One 2015;10:e0119045. 25793718 10.1371/journal.pone.0119045 PMC4368555

[31] Chen H-J Zhan C Cai L-M. White matter microstructural impairments in amyotrophic lateral sclerosis: a mean apparent propagator MRI study. Neuroimage Clin 2021;32:102863. 34700102 10.1016/j.nicl.2021.102863 PMC8551695

[32] Basaia S Filippi M Spinelli EG. White matter microstructure breakdown in the motor neuron disease spectrum: recent advances using diffusion magnetic resonance imaging. Front Neurol 2019;10:193. 30891004 10.3389/fneur.2019.00193 PMC6413536

[33] Bede P Chipika RH Finegan E. Brainstem pathology in amyotrophic lateral sclerosis and primary lateral sclerosis: a longitudinal neuroimaging study. Neuroimage Clin 2019;24:102054. 31711033 10.1016/j.nicl.2019.102054 PMC6849418

[34] Canu E Agosta F Riva N. The topography of brain microstructural damage in amyotrophic lateral sclerosis assessed using diffusion tensor MR imaging. AJNR Am J Neuroradiol 2011;32:1307–1314. 21680655 10.3174/ajnr.A2469 PMC7966037

[35] Zhang F Chen G He M. Altered white matter microarchitecture in amyotrophic lateral sclerosis: a voxel-based meta-analysis of diffusion tensor imaging. Neuroimage Clin 2018;19:122–129. 30035009 10.1016/j.nicl.2018.04.005 PMC6051469

[36] Chiò A Pagani M Agosta F. Neuroimaging in amyotrophic lateral sclerosis: insights into structural and functional changes. Lancet Neurol 2014;13:1228–1240. 25453462 10.1016/S1474-4422(14)70167-X

[37] Verber NS Shepheard SR Sassani M. Biomarkers in motor neuron disease: a state of the art review. Front Neurol 2019;10:291. 31001186 10.3389/fneur.2019.00291 PMC6456669

[38] Li W Wei Q Hou Y. Disruption of the white matter structural network and its correlation with baseline progression rate in patients with sporadic amyotrophic lateral sclerosis. Transl Neurodegener 2021;10:35. 34511130 10.1186/s40035-021-00255-0 PMC8436442

[39] Steinbach R Gaur N Roediger A. Disease aggressiveness signatures of amyotrophic lateral sclerosis in white matter tracts revealed by the D50 disease progression model. Hum Brain Mapp 2021;42:737–752. 33103324 10.1002/hbm.25258 PMC7814763

[40] Trojsi F Caiazzo G Siciliano M. Microstructural correlates of Edinburgh Cognitive and Behavioural ALS Screen (ECAS) changes in amyotrophic lateral sclerosis. Psychiatry Res Neuroimaging 2019;288:67–75. 30987770 10.1016/j.pscychresns.2019.04.001

[41] Kamagata K Andica C Kato A. Diffusion magnetic resonance imaging-based biomarkers for neurodegenerative diseases. Int J Mol Sci 2021;22:5216. 34069159 10.3390/ijms22105216 PMC8155849

[42] Simon NG Turner MR Vucic S. Quantifying disease progression in amyotrophic lateral sclerosis. Ann Neurol 2014;76:643–657. 25223628 10.1002/ana.24273 PMC4305209

[43] Kalra S Müller H-P Ishaque A. A prospective harmonized multicenter DTI study of cerebral white matter degeneration in ALS. Neurology 2020;95:e943–e952. 32646955 10.1212/WNL.0000000000010235 PMC7668555

[44] Keil C Prell T Peschel T. Longitudinal diffusion tensor imaging in amyotrophic lateral sclerosis. BMC Neurosci 2012;13:141. 23134591 10.1186/1471-2202-13-141 PMC3531302

[45] Nandedkar SD Barkhaus PE Stålberg EV. Motor unit number index (MUNIX): principle, method, and findings in healthy subjects and in patients with motor neuron disease. Muscle Nerve 2010;42:798–807. 20976783 10.1002/mus.21824

