# Supplementary material for: Diffusion tensor imaging biomarkers and clinical assessments in amyotrophic lateral sclerosis (ALS) patients: an exploratory study
Source: Ann Med Surg (Lond). 2024 Jul 23;86(9):5080–90. doi: 10.1097/MS9.0000000000002332 (PMC11374192; doi:10.1097/MS9.0000000000002332)
Supplement: Supplementary file 2 [file ms9-86-5080-s002.docx]

| Supplementary Table S5. Significant correlations of AD metrics between pairs of regions | | | |
| --- | --- | --- | --- |
| Correlation Between | | **Correlation Coefficient (R)** | **P-value** |
| Corticospinal_tract_R | Pontine_crossing_tract | 0.760 | 0.007 |
| Cerebral_peduncle_R | Pontine_crossing_tract | 0.603 | 0.049 |
| Posterior_thalamic_radiation_R | Pontine_crossing_tract | -0.702 | 0.016 |
| Hippocampus_L | Body_of_corpus_callosum | 0.652 | 0.030 |
| Cerebral_peduncle_R | Splenium_of_corpus_callosum | -0.706 | 0.015 |
| Posterior_thalamic_radiation_L | Fornix | 0.615 | 0.044 |
| Cingulum_R | Splenium_of_corpus_callosum | 0.669 | 0.024 |
| Hippocampus_R | Splenium_of_corpus_callosum | 0.662 | 0.027 |
| Tapetum_L | Fornix | 0.646 | 0.032 |
| Pontine_crossing_tract | Corticospinal_tract_R | 0.760 | 0.007 |
| Corticospinal_tract_R | Corticospinal_tract_L | 0.714 | 0.014 |
| Inferior_cerebellar_peduncle_R | Corticospinal_tract_R | 0.627 | 0.039 |
| Inferior_cerebellar_peduncle_R | Corticospinal_tract_L | 0.844 | 0.001 |
| Superior_cerebellar_peduncle_L | Corticospinal_tract_R | 0.647 | 0.032 |
| Cerebral_peduncle_R | Corticospinal_tract_R | 0.709 | 0.014 |
| Posterior_thalamic_radiation_R | Corticospinal_tract_R | -0.816 | 0.002 |
| Posterior_thalamic_radiation_R | Corticospinal_tract_L | -0.699 | 0.017 |
| Uncinate_fasciculus_L | Corticospinal_tract_R | 0.618 | 0.043 |
| Tapetum_R | Corticospinal_tract_R | -0.722 | 0.012 |
| Medial_lemniscus_R | Medial_lemniscus_L | 0.742 | 0.009 |
| Superior_longitudinal_fasciculus_L | Medial_lemniscus_R | 0.682 | 0.021 |
| Uncinate_fasciculus_R | Medial_lemniscus_R | 0.674 | 0.023 |
| Uncinate_fasciculus_R | Medial_lemniscus_L | 0.619 | 0.042 |
| Cerebral_peduncle_R | Superior_cerebellar_peduncle_L | 0.657 | 0.028 |
| Posterior_thalamic_radiation_L | Superior_cerebellar_peduncle_L | -0.754 | 0.007 |
| Fornix_cres_Stria_terminalis_R | Superior_cerebellar_peduncle_L | -0.639 | 0.034 |
| Uncinate_fasciculus_L | Superior_cerebellar_peduncle_L | 0.672 | 0.024 |
| Cerebral_peduncle_R | Cerebral_peduncle_L | 0.678 | 0.022 |
| Posterior_thalamic_radiation_R | Cerebral_peduncle_R | -0.664 | 0.026 |
| Cingulum_R | Cerebral_peduncle_R | -0.757 | 0.007 |
| Cingulum_R | Cerebral_peduncle_L | -0.719 | 0.013 |
| Tapetum_R | Cerebral_peduncle_R | -0.627 | 0.039 |
| Anterior_limb_of_internal_capsule_R | Anterior_limb_of_internal_capsule_L | 0.752 | 0.008 |
| Anterior_limb_of_internal_capsule_R | Posterior_limb_of_internal_capsule_R | 0.727 | 0.011 |
| Anterior_limb_of_internal_capsule_R | Anterior_corona_radiata_L | 0.645 | 0.032 |
| Anterior_limb_of_internal_capsule_R | Superior_fronto_occipital_fasciculus_R | 0.900 | 0.00001 |
| Anterior_limb_of_internal_capsule_R | Superior_fronto_occipital_fasciculus_L | 0.736 | 0.010 |
| Anterior_limb_of_internal_capsule_L | Superior_fronto_occipital_fasciculus_R | 0.815 | 0.002 |
| Anterior_limb_of_internal_capsule_L | Superior_fronto_occipital_fasciculus_L | 0.733 | 0.010 |
| Posterior_limb_of_internal_capsule_R | Body_of_corpus_callosum | -0.664 | 0.026 |
| Posterior_limb_of_internal_capsule_R | Posterior_limb_of_internal_capsule_L | 0.655 | 0.029 |
| Posterior_limb_of_internal_capsule_R | Retrolenticular_part_of_internal_capsule_R | 0.755 | 0.007 |
| Posterior_limb_of_internal_capsule_R | Retrolenticular_part_of_internal_capsule_L | 0.645 | 0.032 |
| Posterior_limb_of_internal_capsule_R | Posterior_thalamic_radiation_L | 0.627 | 0.039 |
| Posterior_limb_of_internal_capsule_R | Sagittal_stratum_R | 0.645 | 0.032 |
| Posterior_limb_of_internal_capsule_R | Hippocampus_L | -0.609 | 0.047 |
| Posterior_limb_of_internal_capsule_R | Superior_fronto_occipital_fasciculus_R | 0.718 | 0.013 |
| Posterior_limb_of_internal_capsule_R | Superior_fronto_occipital_fasciculus_L | 0.627 | 0.039 |
| Posterior_limb_of_internal_capsule_L | Pontine_crossing_tract | -0.755 | 0.007 |
| Retrolenticular_part_of_internal_capsule_R | Retrolenticular_part_of_internal_capsule_L | 0.951 | 0.000 |
| Retrolenticular_part_of_internal_capsule_R | Superior_corona_radiata_R | 0.845 | 0.001 |
| Retrolenticular_part_of_internal_capsule_R | Superior_corona_radiata_L | 0.847 | 0.001 |
| Retrolenticular_part_of_internal_capsule_R | Posterior_corona_radiata_L | 0.678 | 0.022 |
| Retrolenticular_part_of_internal_capsule_R | Sagittal_stratum_R | 0.819 | 0.002 |
| Retrolenticular_part_of_internal_capsule_R | Sagittal_stratum_L | 0.631 | 0.037 |
| Retrolenticular_part_of_internal_capsule_L | Superior_corona_radiata_L | 0.618 | 0.043 |
| Retrolenticular_part_of_internal_capsule_L | Posterior_corona_radiata_L | 0.623 | 0.041 |
| Retrolenticular_part_of_internal_capsule_L | Posterior_thalamic_radiation_R | 0.652 | 0.030 |
| Retrolenticular_part_of_internal_capsule_L | Sagittal_stratum_R | 0.804 | 0.003 |
| Anterior_corona_radiata_R | Anterior_corona_radiata_L | 0.918 | 0.000 |
| Anterior_corona_radiata_R | Superior_corona_radiata_R | 0.618 | 0.043 |
| Anterior_corona_radiata_R | Superior_corona_radiata_L | 0.782 | 0.004 |
| Anterior_corona_radiata_L | Superior_corona_radiata_L | 0.682 | 0.021 |
| Posterior_corona_radiata_L | Sagittal_stratum_R | 0.614 | 0.044 |
| Posterior_thalamic_radiation_R | Uncinate_fasciculus_L | -0.729 | 0.011 |
| Posterior_thalamic_radiation_L | Sagittal_stratum_R | 0.622 | 0.041 |
| Posterior_thalamic_radiation_L | Sagittal_stratum_L | 0.867 | 0.001 |
| Posterior_thalamic_radiation_L | Tapetum_L | 0.688 | 0.019 |
| Sagittal_stratum_R | Sagittal_stratum_L | 0.746 | 0.007 |
| Sagittal_stratum_R | Fornix_cres_Stria_terminalis_L | 0.765 | 0.006 |
| Cingulum_R | Cingulum_L | 0.655 | 0.029 |
| Fornix_cres_Stria_terminalis_R | Posterior_thalamic_radiation_L | 0.732 | 0.011 |
| Fornix_cres_Stria_terminalis_R | Tapetum_L | 0.734 | 0.010 |
| Tapetum_R | Tapetum_L | 0.692 | 0.018 |
| Middle_cerebellar_peduncle | Anterior_corona_radiata_L | 0.682 | 0.021 |
| Genu_of_corpus_callosum | Superior_fronto_occipital_fasciculus_R | 0.645 | 0.032 |
| Medial_lemniscus_L | Superior_longitudinal_fasciculus_R | 0.618 | 0.043 |
| Retrolenticular_part_of_internal_capsule_R | External_capsule_L | 0.718 | 0.013 |
| Retrolenticular_part_of_internal_capsule_L | Superior_fronto_occipital_fasciculus_R | 0.673 | 0.023 |
| Anterior_corona_radiata_R | Superior_fronto_occipital_fasciculus_L | 0.836 | 0.001 |
| Anterior_corona_radiata_L | Superior_fronto_occipital_fasciculus_L | 0.845 | 0.001 |
| Superior_corona_radiata_R | Superior_corona_radiata_L | 0.609 | 0.047 |
| Superior_corona_radiata_R | External_capsule_L | 0.664 | 0.026 |
| Superior_corona_radiata_L | External_capsule_L | 0.609 | 0.047 |
| Superior_corona_radiata_L | Superior_fronto_occipital_fasciculus_L | 0.691 | 0.019 |
| Posterior_corona_radiata_R | Superior_corona_radiata_R | 0.682 | 0.021 |
| Posterior_corona_radiata_R | External_capsule_R | 0.627 | 0.039 |
| Posterior_corona_radiata_R | External_capsule_L | 0.745 | 0.008 |
| Posterior_corona_radiata_R | Superior_longitudinal_fasciculus_L | 0.718 | 0.013 |
| Posterior_thalamic_radiation_R | Superior_longitudinal_fasciculus_R | 0.682 | 0.021 |
| Posterior_thalamic_radiation_L | Posterior_limb_of_internal_capsule_R | 0.627 | 0.039 |
| Sagittal_stratum_R | Posterior_limb_of_internal_capsule_R | 0.645 | 0.032 |
| Sagittal_stratum_R | Anterior_corona_radiata_R | 0.791 | 0.004 |
| Sagittal_stratum_R | Anterior_corona_radiata_L | 0.800 | 0.003 |
| Sagittal_stratum_R | External_capsule_R | 0.664 | 0.026 |
| Sagittal_stratum_R | External_capsule_L | 0.764 | 0.006 |
| Sagittal_stratum_R | Superior_fronto_occipital_fasciculus_L | 0.655 | 0.029 |
| External_capsule_R | External_capsule_L | 0.664 | 0.026 |
| External_capsule_L | Superior_longitudinal_fasciculus_L | 0.691 | 0.019 |
| Cingulum_R | Anterior_corona_radiata_R | -0.609 | 0.047 |
| Cingulum_R | Superior_longitudinal_fasciculus_R | 0.691 | 0.019 |
| Cingulum_L | Superior_longitudinal_fasciculus_R | 0.618 | 0.043 |
| Fornix_cres_Stria_terminalis_L | Anterior_corona_radiata_R | 0.691 | 0.019 |
| Fornix_cres_Stria_terminalis_L | Anterior_corona_radiata_L | 0.673 | 0.023 |
| Fornix_cres_Stria_terminalis_L | External_capsule_R | 0.764 | 0.006 |
| Fornix_cres_Stria_terminalis_L | Superior_fronto_occipital_fasciculus_L | 0.618 | 0.043 |
| Uncinate_fasciculus_L | Superior_longitudinal_fasciculus_R | -0.727 | 0.011 |
| Tapetum_R | Superior_corona_radiata_R | -0.664 | 0.026 |
| Tapetum_R | Superior_corona_radiata_L | -0.691 | 0.019 |

| Supplementary Table S6. Significant correlations of MD metrics between pairs of regions | | | |
| --- | --- | --- | --- |
| Correlation Between | | **Correlation Coefficient (R)** | **P-value** |
| Pontine_crossing_tract | Corticospinal_tract_R | 0.724 | 0.012 |
| Pontine_crossing_tract | Corticospinal_tract_L | 0.714 | 0.014 |
| Pontine_crossing_tract | Posterior_thalamic_radiation_R | -0.705 | 0.015 |
| Genu_of_corpus_callosum | Inferior_cerebellar_peduncle_R | -0.721 | 0.012 |
| Splenium_of_corpus_callosum | Cerebral_peduncle_R | -0.718 | 0.013 |
| Splenium_of_corpus_callosum | Cerebral_peduncle_L | -0.708 | 0.015 |
| Splenium_of_corpus_callosum | Hippocampus_R | 0.648 | 0.031 |
| Fornix | Medial_lemniscus_L | -0.649 | 0.031 |
| Corticospinal_tract_R | Corticospinal_tract_L | 0.829 | 0.002 |
| Corticospinal_tract_R | Cerebral_peduncle_R | 0.734 | 0.010 |
| Corticospinal_tract_R | Posterior_thalamic_radiation_R | -0.705 | 0.015 |
| Corticospinal_tract_R | Fornix_cres_Stria_terminalis_R | -0.713 | 0.014 |
| Corticospinal_tract_R | Tapetum_L | -0.633 | 0.037 |
| Corticospinal_tract_L | Inferior_cerebellar_peduncle_R | 0.698 | 0.017 |
| Corticospinal_tract_L | Posterior_thalamic_radiation_R | -0.660 | 0.027 |
| Medial_lemniscus_R | Cerebral_peduncle_L | 0.655 | 0.026 |
| Medial_lemniscus_R | Posterior_corona_radiata_R | 0.699 | 0.017 |
| Medial_lemniscus_R | Uncinate_fasciculus_R | 0.704 | 0.016 |
| Medial_lemniscus_L | Cingulum_R | 0.770 | 0.006 |
| Medial_lemniscus_L | Cingulum_L | 0.779 | 0.005 |
| Medial_lemniscus_L | Uncinate_fasciculus_R | 0.713 | 0.014 |
| Medial_lemniscus_L | Uncinate_fasciculus_L | 0.785 | 0.004 |
| Inferior_cerebellar_peduncle_R | Posterior_thalamic_radiation_R | -0.638 | 0.035 |
| Inferior_cerebellar_peduncle_R | Sagittal_stratum_L | -0.611 | 0.046 |
| Cerebral_peduncle_R | Cerebral_peduncle_L | 0.805 | 0.003 |
| Cerebral_peduncle_R | Fornix_cres_Stria_terminalis_R | -0.682 | 0.021 |
| Cerebral_peduncle_R | Uncinate_fasciculus_L | 0.631 | 0.037 |
| Retrolenticular_part_of_internal_capsule_R | Posterior_corona_radiata_R | 0.767 | 0.006 |
| Retrolenticular_part_of_internal_capsule_R | Posterior_corona_radiata_L | 0.872 | 0.000 |
| Retrolenticular_part_of_internal_capsule_R | Posterior_thalamic_radiation_R | 0.773 | 0.005 |
| Retrolenticular_part_of_internal_capsule_R | Sagittal_stratum_R | 0.786 | 0.004 |
| Retrolenticular_part_of_internal_capsule_R | Sagittal_stratum_L | 0.655 | 0.029 |
| Retrolenticular_part_of_internal_capsule_R | Uncinate_fasciculus_R | 0.845 | 0.001 |
| Retrolenticular_part_of_internal_capsule_R | Uncinate_fasciculus_L | 0.677 | 0.022 |
| Posterior_corona_radiata_R | Posterior_corona_radiata_L | 0.835 | 0.001 |
| Posterior_corona_radiata_R | Uncinate_fasciculus_R | 0.676 | 0.022 |
| Posterior_corona_radiata_L | Sagittal_stratum_R | 0.862 | 0.001 |
| Posterior_corona_radiata_L | Sagittal_stratum_L | 0.659 | 0.027 |
| Posterior_corona_radiata_L | Uncinate_fasciculus_R | 0.677 | 0.022 |
| Posterior_corona_radiata_L | Uncinate_fasciculus_L | 0.664 | 0.026 |
| Posterior_thalamic_radiation_L | Sagittal_stratum_L | 0.811 | 0.002 |
| Posterior_thalamic_radiation_L | Fornix_cres_Stria_terminalis_R | 0.647 | 0.032 |
| Sagittal_stratum_R | Sagittal_stratum_L | 0.676 | 0.022 |
| Sagittal_stratum_R | Uncinate_fasciculus_R | 0.605 | 0.049 |
| Sagittal_stratum_R | Uncinate_fasciculus_L | 0.785 | 0.004 |
| Sagittal_stratum_L | Sagittal_stratum_R | 0.676 | 0.022 |
| Cingulum_R | Cingulum_L | 0.934 | 0.000 |
| Cingulum_R | Uncinate_fasciculus_L | 0.715 | 0.013 |
| Cingulum_L | Uncinate_fasciculus_L | 0.641 | 0.034 |
| Fornix_cres_Stria_terminalis_R | Tapetum_L | 0.803 | 0.003 |
| Uncinate_fasciculus_R | Uncinate_fasciculus_L | 0.710 | 0.014 |
| Pontine_crossing_tract | Tapetum_R | -0.609 | 0.047 |
| Genu_of_corpus_callosum | Superior_fronto_occipital_fasciculus_R | 0.627 | 0.039 |
| Splenium_of_corpus_callosum | Superior_cerebellar_peduncle_L | -0.864 | 0.001 |
| Splenium_of_corpus_callosum | Superior_fronto_occipital_fasciculus_L | 0.609 | 0.047 |
| Corticospinal_tract_R | Tapetum_R | -0.755 | 0.007 |
| Medial_lemniscus_L | Superior_cerebellar_peduncle_L | 0.618 | 0.043 |
| Medial_lemniscus_L | External_capsule_R | 0.627 | 0.039 |
| Inferior_cerebellar_peduncle_R | Superior_fronto_occipital_fasciculus_L | -0.691 | 0.019 |
| Cerebral_peduncle_R | Superior_cerebellar_peduncle_L | 0.609 | 0.047 |
| Cerebral_peduncle_R | Tapetum_R | -0.727 | 0.011 |
| Anterior_limb_of_internal_capsule_R | Anterior_limb_of_internal_capsule_L | 0.827 | 0.002 |
| Anterior_limb_of_internal_capsule_R | Posterior_limb_of_internal_capsule_R | 0.764 | 0.006 |
| Anterior_limb_of_internal_capsule_R | Posterior_limb_of_internal_capsule_L | 0.718 | 0.013 |
| Anterior_limb_of_internal_capsule_R | Retrolenticular_part_of_internal_capsule_L | 0.664 | 0.026 |
| Anterior_limb_of_internal_capsule_R | Anterior_corona_radiata_L | 0.800 | 0.003 |
| Anterior_limb_of_internal_capsule_R | Superior_corona_radiata_R | 0.945 | 0.000 |
| Anterior_limb_of_internal_capsule_R | Superior_corona_radiata_L | 0.864 | 0.001 |
| Anterior_limb_of_internal_capsule_R | Posterior_corona_radiata_R | 0.827 | 0.002 |
| Anterior_limb_of_internal_capsule_R | External_capsule_R | 0.791 | 0.004 |
| Anterior_limb_of_internal_capsule_R | External_capsule_L | 0.791 | 0.004 |
| Anterior_limb_of_internal_capsule_R | Superior_longitudinal_fasciculus_R | 0.718 | 0.013 |
| Anterior_limb_of_internal_capsule_R | Superior_longitudinal_fasciculus_L | 0.855 | 0.001 |
| Anterior_limb_of_internal_capsule_L | Posterior_limb_of_internal_capsule_L | 0.618 | 0.043 |
| Anterior_limb_of_internal_capsule_L | Anterior_corona_radiata_L | 0.782 | 0.004 |
| Anterior_limb_of_internal_capsule_L | Superior_corona_radiata_R | 0.745 | 0.008 |
| Anterior_limb_of_internal_capsule_L | Superior_corona_radiata_L | 0.736 | 0.010 |
| Anterior_limb_of_internal_capsule_L | Superior_longitudinal_fasciculus_L | 0.618 | 0.043 |
| Anterior_limb_of_internal_capsule_L | Superior_fronto_occipital_fasciculus_R | 0.636 | 0.035 |
| Anterior_limb_of_internal_capsule_L | Superior_fronto_occipital_fasciculus_L | 0.782 | 0.004 |
| Posterior_limb_of_internal_capsule_R | Posterior_limb_of_internal_capsule_L | 0.809 | 0.003 |
| Posterior_limb_of_internal_capsule_R | Retrolenticular_part_of_internal_capsule_L | 0.836 | 0.001 |
| Posterior_limb_of_internal_capsule_R | Superior_corona_radiata_R | 0.636 | 0.035 |
| Posterior_limb_of_internal_capsule_R | Posterior_corona_radiata_R | 0.609 | 0.047 |
| Posterior_limb_of_internal_capsule_L | Retrolenticular_part_of_internal_capsule_L | 0.627 | 0.039 |
| Retrolenticular_part_of_internal_capsule_R | Anterior_limb_of_internal_capsule_R | 0.855 | 0.001 |
| Retrolenticular_part_of_internal_capsule_R | Anterior_limb_of_internal_capsule_L | 0.773 | 0.005 |
| Retrolenticular_part_of_internal_capsule_R | Posterior_limb_of_internal_capsule_R | 0.700 | 0.016 |
| Retrolenticular_part_of_internal_capsule_R | Retrolenticular_part_of_internal_capsule_L | 0.782 | 0.004 |
| Retrolenticular_part_of_internal_capsule_R | Anterior_corona_radiata_L | 0.764 | 0.006 |
| Retrolenticular_part_of_internal_capsule_R | Superior_corona_radiata_R | 0.791 | 0.004 |
| Retrolenticular_part_of_internal_capsule_R | Superior_corona_radiata_L | 0.800 | 0.003 |
| Retrolenticular_part_of_internal_capsule_R | Posterior_corona_radiata_R | 0.800 | 0.003 |
| Retrolenticular_part_of_internal_capsule_R | External_capsule_R | 0.709 | 0.015 |
| Retrolenticular_part_of_internal_capsule_R | External_capsule_L | 0.800 | 0.003 |
| Retrolenticular_part_of_internal_capsule_R | Superior_longitudinal_fasciculus_R | 0.609 | 0.047 |
| Retrolenticular_part_of_internal_capsule_R | Superior_longitudinal_fasciculus_L | 0.827 | 0.002 |
| Retrolenticular_part_of_internal_capsule_R | Superior_fronto_occipital_fasciculus_L | 0.645 | 0.032 |
| Retrolenticular_part_of_internal_capsule_L | Anterior_corona_radiata_L | 0.645 | 0.032 |
| Retrolenticular_part_of_internal_capsule_L | Superior_corona_radiata_R | 0.636 | 0.035 |
| Retrolenticular_part_of_internal_capsule_L | Superior_corona_radiata_L | 0.655 | 0.029 |
| Retrolenticular_part_of_internal_capsule_L | Posterior_corona_radiata_R | 0.618 | 0.043 |
| Anterior_corona_radiata_R | Anterior_limb_of_internal_capsule_R | 0.718 | 0.013 |
| Anterior_corona_radiata_R | Anterior_limb_of_internal_capsule_L | 0.755 | 0.007 |
| Anterior_corona_radiata_R | Retrolenticular_part_of_internal_capsule_L | 0.655 | 0.029 |
| Anterior_corona_radiata_R | Anterior_corona_radiata_L | 0.918 | 0.000 |
| Anterior_corona_radiata_R | Superior_corona_radiata_R | 0.718 | 0.013 |
| Anterior_corona_radiata_R | Superior_corona_radiata_L | 0.791 | 0.004 |
| Anterior_corona_radiata_R | Posterior_corona_radiata_R | 0.655 | 0.029 |
| Anterior_corona_radiata_R | External_capsule_L | 0.655 | 0.029 |
| Anterior_corona_radiata_R | Superior_longitudinal_fasciculus_R | 0.627 | 0.039 |
| Anterior_corona_radiata_R | Superior_longitudinal_fasciculus_L | 0.782 | 0.004 |
| Anterior_corona_radiata_R | Tapetum_R | -0.691 | 0.019 |
| Anterior_corona_radiata_L | Superior_corona_radiata_R | 0.864 | 0.001 |
| Anterior_corona_radiata_L | Superior_corona_radiata_L | 0.818 | 0.002 |
| Anterior_corona_radiata_L | Posterior_corona_radiata_R | 0.664 | 0.026 |
| Anterior_corona_radiata_L | External_capsule_R | 0.709 | 0.015 |
| Anterior_corona_radiata_L | External_capsule_L | 0.673 | 0.023 |
| Anterior_corona_radiata_L | Superior_longitudinal_fasciculus_R | 0.773 | 0.005 |
| Anterior_corona_radiata_L | Superior_longitudinal_fasciculus_L | 0.818 | 0.022 |
| Anterior_corona_radiata_L | Superior_fronto_occipital_fasciculus_R | 0.645 | 0.032 |
| Anterior_corona_radiata_L | Superior_fronto_occipital_fasciculus_L | 0.691 | 0.019 |
| Superior_corona_radiata_R | Superior_corona_radiata_L | 0.918 | 0.000 |
| Superior_corona_radiata_R | Posterior_corona_radiata_R | 0.836 | 0.001 |
| Superior_corona_radiata_R | External_capsule_R | 0.809 | 0.003 |
| Superior_corona_radiata_R | External_capsule_L | 0.809 | 0.003 |
| Superior_corona_radiata_R | Superior_longitudinal_fasciculus_R | 0.827 | 0.002 |
| Superior_corona_radiata_R | Superior_longitudinal_fasciculus_L | 0.864 | 0.001 |
| Superior_corona_radiata_R | Superior_fronto_occipital_fasciculus_L | 0.645 | 0.032 |
| Superior_corona_radiata_L | Posterior_corona_radiata_R | 0.873 | 0.000 |
| Superior_corona_radiata_L | External_capsule_R | 0.645 | 0.032 |
| Superior_corona_radiata_L | External_capsule_L | 0.782 | 0.004 |
| Superior_corona_radiata_L | Superior_longitudinal_fasciculus_R | 0.736 | 0.010 |
| Superior_corona_radiata_L | Superior_longitudinal_fasciculus_L | 0.845 | 0.001 |
| Superior_corona_radiata_L | Superior_fronto_occipital_fasciculus_L | 0.627 | 0.039 |
| Posterior_corona_radiata_R | External_capsule_R | 0.745 | 0.008 |
| Posterior_corona_radiata_R | External_capsule_L | 0.882 | 0.000 |
| Posterior_corona_radiata_R | Superior_longitudinal_fasciculus_R | 0.818 | 0.002 |
| Posterior_corona_radiata_R | Superior_longitudinal_fasciculus_L | 0.945 | 0.000 |
| Posterior_corona_radiata_L | Anterior_limb_of_internal_capsule_R | 0.773 | 0.005 |
| Posterior_corona_radiata_L | Anterior_limb_of_internal_capsule_L | 0.800 | 0.003 |
| Posterior_corona_radiata_L | Retrolenticular_part_of_internal_capsule_L | 0.664 | 0.026 |
| Posterior_corona_radiata_L | Anterior_corona_radiata_L | 0.745 | 0.008 |
| Posterior_corona_radiata_L | Superior_corona_radiata_R | 0.700 | 0.016 |
| Posterior_corona_radiata_L | Superior_corona_radiata_L | 0.809 | 0.003 |
| Posterior_corona_radiata_L | Posterior_corona_radiata_R | 0.791 | 0.004 |
| Posterior_corona_radiata_L | External_capsule_R | 0.664 | 0.026 |
| Posterior_corona_radiata_L | External_capsule_L | 0.809 | 0.003 |
| Posterior_corona_radiata_L | Superior_longitudinal_fasciculus_L | 0.836 | 0.001 |
| Posterior_thalamic_radiation_R | Superior_fronto_occipital_fasciculus_L | 0.636 | 0.035 |
| Posterior_thalamic_radiation_L | Superior_cerebellar_peduncle_L | -0.727 | 0.011 |
| Posterior_thalamic_radiation_L | Anterior_limb_of_internal_capsule_L | 0.764 | 0.006 |
| Posterior_thalamic_radiation_L | Superior_fronto_occipital_fasciculus_L | 0.800 | 0.003 |
| Sagittal_stratum_R | Anterior_corona_radiata_L | 0.691 | 0.019 |
| Sagittal_stratum_R | External_capsule_R | 0.700 | 0.016 |
| Sagittal_stratum_R | External_capsule_L | 0.709 | 0.015 |
| Sagittal_stratum_R | Superior_longitudinal_fasciculus_L | 0.636 | 0.035 |
| Sagittal_stratum_L | Anterior_limb_of_internal_capsule_L | 0.736 | 0.010 |
| Sagittal_stratum_L | Superior_fronto_occipital_fasciculus_L | 0.700 | 0.016 |
| External_capsule_R | External_capsule_L | 0.927 | 0.000 |
| External_capsule_R | Superior_longitudinal_fasciculus_R | 0.709 | 0.015 |
| External_capsule_R | Superior_longitudinal_fasciculus_L | 0.827 | 0.002 |
| External_capsule_L | Superior_longitudinal_fasciculus_R | 0.682 | 0.021 |
| External_capsule_L | Superior_longitudinal_fasciculus_L | 0.882 | 0.000 |
| Cingulum_R | External_capsule_R | 0.809 | 0.003 |
| Cingulum_R | External_capsule_L | 0.700 | 0.016 |
| Cingulum_L | External_capsule_R | 0.691 | 0.019 |
| Cingulum_L | Superior_longitudinal_fasciculus_R | 0.691 | 0.019 |
| Fornix_cres_Stria_terminalis_R | Superior_cerebellar_peduncle_L | -0.827 | 0.002 |
| Fornix_cres_Stria_terminalis_R | Superior_fronto_occipital_fasciculus_L | 0.664 | 0.026 |
| Fornix_cres_Stria_terminalis_L | Superior_corona_radiata_L | 0.618 | 0.043 |
| Fornix_cres_Stria_terminalis_L | Superior_fronto_occipital_fasciculus_R | 0.818 | 0.002 |
| Fornix_cres_Stria_terminalis_L | Superior_fronto_occipital_fasciculus_L | 0.682 | 0.021 |
| Superior_longitudinal_fasciculus_R | Superior_longitudinal_fasciculus_L | 0.873 | 0.000 |
| Superior_fronto_occipital_fasciculus_R | Superior_fronto_occipital_fasciculus_L | 0.736 | 0.010 |
| Uncinate_fasciculus_R | Superior_cerebellar_peduncle_L | 0.618 | 0.043 |
| Uncinate_fasciculus_L | External_capsule_R | 0.655 | 0.029 |
| Uncinate_fasciculus_L | Superior_longitudinal_fasciculus_L | 0.609 | 0.047 |
| Tapetum_L | Superior_cerebellar_peduncle_L | -0.618 | 0.043 |
| Tapetum_L | Tapetum_R | 0.655 | 0.029 |

| Supplementary Table S7. Significant correlations of RD metrics between pairs of regions | | | |
| --- | --- | --- | --- |
| Correlation Between | | **Correlation Coefficient (R)** | **P-value** |
| Pontine_crossing_tract | Corticospinal_tract_R | 0.705 | 0.015 |
| Pontine_crossing_tract | Corticospinal_tract_L | 0.768 | 0.006 |
| Genu_of_corpus_callosum | Inferior_cerebellar_peduncle_R | -0.744 | 0.009 |
| Genu_of_corpus_callosum | Posterior_thalamic_radiation_R | 0.707 | 0.420 |
| Body_of_corpus_callosum | Fornix_cres_Stria_terminalis_L | 0.649 | 0.031 |
| Corticospinal_tract_R | Corticospinal_tract_L | 0.858 | 0.001 |
| Corticospinal_tract_R | Cerebral_peduncle_R | -0.726 | 0.011 |
| Corticospinal_tract_R | Fornix_cres_Stria_terminalis_R | -0.702 | 0.016 |
| Corticospinal_tract_R | Tapetum_R | -0.701 | 0.016 |
| Corticospinal_tract_R | Tapetum_L | -0.628 | 0.039 |
| Corticospinal_tract_L | Inferior_cerebellar_peduncle_R | 0.606 | 0.048 |
| Medial_lemniscus_R | Cerebral_peduncle_L | 0.698 | 0.017 |
| Medial_lemniscus_R | Posterior_corona_radiata_R | 0.638 | 0.035 |
| Medial_lemniscus_R | Cingulum_R | 0.679 | 0.022 |
| Medial_lemniscus_R | Tapetum_R | -0.628 | 0.038 |
| Medial_lemniscus_R | Tapetum_L | -0.656 | 0.028 |
| Medial_lemniscus_L | Sagittal_stratum_R | 0.664 | 0.026 |
| Medial_lemniscus_L | Cingulum_R | 0.767 | 0.006 |
| Medial_lemniscus_L | Cingulum_L | 0.871 | 0.000 |
| Medial_lemniscus_L | Fornix_cres_Stria_terminalis_R | -0.652 | 0.030 |
| Medial_lemniscus_L | Uncinate_fasciculus_R | 0.657 | 0.028 |
| Medial_lemniscus_L | Uncinate_fasciculus_L | 0.633 | 0.037 |
| Medial_lemniscus_L | Tapetum_R | -0.606 | 0.048 |
| Medial_lemniscus_L | Tapetum_L | -0.703 | 0.016 |
| Inferior_cerebellar_peduncle_R | Posterior_thalamic_radiation_R | -0.651 | 0.030 |
| Inferior_cerebellar_peduncle_R | Posterior_thalamic_radiation_L | -0.616 | 0.043 |
| Inferior_cerebellar_peduncle_L | Superior_cerebellar_peduncle_R | 0.677 | 0.022 |
| Cerebral_peduncle_R | Cerebral_peduncle_L | 0.859 | 0.001 |
| Cerebral_peduncle_R | Cingulum_R | 0.786 | 0.004 |
| Cerebral_peduncle_R | Fornix_cres_Stria_terminalis_R | -0.744 | 0.009 |
| Cerebral_peduncle_R | Tapetum_R | -0.804 | 0.003 |
| Cerebral_peduncle_L | Cingulum_R | 0.766 | 0.006 |
| Cerebral_peduncle_L | Fornix_cres_Stria_terminalis_R | -0.610 | 0.046 |
| Cerebral_peduncle_L | Tapetum_R | -0.641 | 0.034 |
| Posterior_corona_radiata_R | Posterior_corona_radiata_L | 0.831 | 0.002 |
| Posterior_corona_radiata_R | Posterior_thalamic_radiation_R | 0.689 | 0.019 |
| Posterior_corona_radiata_R | Uncinate_fasciculus_R | 0.753 | 0.007 |
| Posterior_corona_radiata_R | Uncinate_fasciculus_L | 0.723 | 0.012 |
| Posterior_corona_radiata_L | Posterior_thalamic_radiation_R | 0.673 | 0.023 |
| Posterior_corona_radiata_L | Sagittal_stratum_R | 0.703 | 0.016 |
| Posterior_corona_radiata_L | Cingulum_R | 0.616 | 0.043 |
| Posterior_corona_radiata_L | Uncinate_fasciculus_R | 0.633 | 0.036 |
| Posterior_corona_radiata_L | Uncinate_fasciculus_L | 0.675 | 0.023 |
| Posterior_thalamic_radiation_R | Posterior_thalamic_radiation_L | 0.660 | 0.027 |
| Posterior_thalamic_radiation_R | Uncinate_fasciculus_L | 0.629 | 0.038 |
| Sagittal_stratum_R | Cingulum_R | 0.749 | 0.008 |
| Sagittal_stratum_R | Cingulum_L | 0.677 | 0.022 |
| Sagittal_stratum_R | Uncinate_fasciculus_L | 0.706 | 0.015 |
| Cingulum_R | Cingulum_L | 0.671 | 0.024 |
| Cingulum_R | Fornix_cres_Stria_terminalis_R | -0.726 | 0.011 |
| Cingulum_R | Uncinate_fasciculus_R | 0.626 | 0.039 |
| Cingulum_R | Tapetum_R | -0.848 | 0.001 |
| Cingulum_L | Uncinate_fasciculus_L | 0.736 | 0.010 |
| Cingulum_L | Tapetum_R | -0.622 | 0.041 |
| Cingulum_L | Tapetum_L | -0.648 | 0.031 |
| Fornix_cres_Stria_terminalis_R | Tapetum_R | 0.669 | 0.024 |
| Fornix_cres_Stria_terminalis_R | Tapetum_L | 0.818 | 0.002 |
| Uncinate_fasciculus_R | Uncinate_fasciculus_L | 0.723 | 0.012 |
| Tapetum_R | Tapetum_L | 0.773 | 0.005 |
| Middle_cerebellar_peduncle | Anterior_limb_of_internal_capsule_L | 0.618 | 0.043 |
| Splenium_of_corpus_callosum | Superior_cerebellar_peduncle_L | -0.664 | 0.026 |
| Splenium_of_corpus_callosum | Superior_fronto_occipital_fasciculus_L | 0.662 | 0.026 |
| Medial_lemniscus_R | Superior_longitudinal_fasciculus_L | 0.664 | 0.026 |
| Medial_lemniscus_L | Superior_cerebellar_peduncle_L | 0.673 | 0.023 |
| Medial_lemniscus_L | External_capsule_R | 0.764 | 0.006 |
| Inferior_cerebellar_peduncle_R | Sagittal_stratum_L | -0.691 | 0.019 |
| Inferior_cerebellar_peduncle_R | Superior_fronto_occipital_fasciculus_L | -0.873 | 0.000 |
| Cerebral_peduncle_R | Superior_cerebellar_peduncle_L | 0.645 | 0.032 |
| Cerebral_peduncle_R | Retrolenticular_part_of_internal_capsule_L | 0.627 | 0.039 |
| Anterior_limb_of_internal_capsule_R | Anterior_limb_of_internal_capsule_L | 0.727 | 0.011 |
| Anterior_limb_of_internal_capsule_R | Posterior_limb_of_internal_capsule_R | 0.791 | 0.004 |
| Anterior_limb_of_internal_capsule_R | Retrolenticular_part_of_internal_capsule_R | 0.809 | 0.003 |
| Anterior_limb_of_internal_capsule_R | Retrolenticular_part_of_internal_capsule_L | 0.818 | 0.002 |
| Anterior_limb_of_internal_capsule_R | Anterior_corona_radiata_R | 0.727 | 0.011 |
| Anterior_limb_of_internal_capsule_R | Anterior_corona_radiata_L | 0.836 | 0.001 |
| Anterior_limb_of_internal_capsule_R | Superior_corona_radiata_R | 0.755 | 0.007 |
| Anterior_limb_of_internal_capsule_R | Superior_corona_radiata_L | 0.764 | 0.006 |
| Anterior_limb_of_internal_capsule_R | External_capsule_R | 0.682 | 0.021 |
| Anterior_limb_of_internal_capsule_R | External_capsule_L | 0.673 | 0.023 |
| Anterior_limb_of_internal_capsule_R | Superior_longitudinal_fasciculus_R | 0.655 | 0.029 |
| Anterior_limb_of_internal_capsule_R | Superior_longitudinal_fasciculus_L | 0.682 | 0.021 |
| Anterior_limb_of_internal_capsule_L | Retrolenticular_part_of_internal_capsule_R | 0.700 | 0.016 |
| Anterior_limb_of_internal_capsule_L | Anterior_corona_radiata_R | 0.736 | 0.010 |
| Anterior_limb_of_internal_capsule_L | Anterior_corona_radiata_L | 0.636 | 0.035 |
| Anterior_limb_of_internal_capsule_L | Superior_corona_radiata_R | 0.764 | 0.006 |
| Anterior_limb_of_internal_capsule_L | Superior_corona_radiata_L | 0.736 | 0.010 |
| Anterior_limb_of_internal_capsule_L | Sagittal_stratum_L | 0.682 | 0.021 |
| Anterior_limb_of_internal_capsule_L | Superior_longitudinal_fasciculus_R | 0.618 | 0.043 |
| Anterior_limb_of_internal_capsule_L | Superior_fronto_occipital_fasciculus_R | 0.664 | 0.026 |
| Anterior_limb_of_internal_capsule_L | Superior_fronto_occipital_fasciculus_L | 0.691 | 0.019 |
| Posterior_limb_of_internal_capsule_R | Retrolenticular_part_of_internal_capsule_L | 0.691 | 0.019 |
| Posterior_limb_of_internal_capsule_L | Retrolenticular_part_of_internal_capsule_R | 0.836 | 0.001 |
| Posterior_limb_of_internal_capsule_L | Anterior_corona_radiata_R | 0.664 | 0.026 |
| Posterior_limb_of_internal_capsule_L | Superior_corona_radiata_R | 0.618 | 0.043 |
| Retrolenticular_part_of_internal_capsule_R | Retrolenticular_part_of_internal_capsule_L | 0.791 | 0.004 |
| Retrolenticular_part_of_internal_capsule_R | Anterior_corona_radiata_R | 0.836 | 0.001 |
| Retrolenticular_part_of_internal_capsule_R | Anterior_corona_radiata_L | 0.818 | 0.002 |
| Retrolenticular_part_of_internal_capsule_R | Superior_corona_radiata_R | 0.664 | 0.026 |
| Retrolenticular_part_of_internal_capsule_R | Superior_corona_radiata_L | 0.673 | 0.023 |
| Retrolenticular_part_of_internal_capsule_R | Sagittal_stratum_L | 0.645 | 0.032 |
| Retrolenticular_part_of_internal_capsule_R | External_capsule_R | 0.727 | 0.011 |
| Retrolenticular_part_of_internal_capsule_R | Superior_longitudinal_fasciculus_R | 0.655 | 0.029 |
| Retrolenticular_part_of_internal_capsule_R | Superior_longitudinal_fasciculus_L | 0.782 | 0.004 |
| Retrolenticular_part_of_internal_capsule_L | Anterior_corona_radiata_R | 0.718 | 0.013 |
| Retrolenticular_part_of_internal_capsule_L | Anterior_corona_radiata_L | 0.664 | 0.026 |
| Retrolenticular_part_of_internal_capsule_L | Superior_longitudinal_fasciculus_L | 0.673 | 0.023 |
| Anterior_corona_radiata_R | Anterior_corona_radiata_L | 0.836 | 0.001 |
| Anterior_corona_radiata_R | Superior_corona_radiata_R | 0.664 | 0.026 |
| Anterior_corona_radiata_R | Superior_corona_radiata_L | 0.782 | 0.004 |
| Anterior_corona_radiata_R | Sagittal_stratum_L | 0.618 | 0.043 |
| Anterior_corona_radiata_R | External_capsule_R | 0.655 | 0.029 |
| Anterior_corona_radiata_R | Superior_longitudinal_fasciculus_R | 0.836 | 0.001 |
| Anterior_corona_radiata_R | Superior_longitudinal_fasciculus_L | 0.909 | 0.00 |
| Anterior_corona_radiata_L | Superior_corona_radiata_L | 0.809 | 0.003 |
| Anterior_corona_radiata_L | External_capsule_R | 0.782 | 0.004 |
| Anterior_corona_radiata_L | External_capsule_L | 0.664 | 0.026 |
| Anterior_corona_radiata_L | Superior_longitudinal_fasciculus_R | 0.864 | 0.001 |
| Anterior_corona_radiata_L | Superior_longitudinal_fasciculus_L | 0.873 | 0.000 |
| Superior_corona_radiata_R | Anterior_corona_radiata_L | 0.745 | 0.008 |
| Superior_corona_radiata_R | Superior_corona_radiata_L | 0.936 | 0.000 |
| Superior_corona_radiata_R | External_capsule_L | 0.682 | 0.021 |
| Superior_corona_radiata_R | Superior_longitudinal_fasciculus_R | 0.809 | 0.003 |
| Superior_corona_radiata_R | Superior_fronto_occipital_fasciculus_R | 0.627 | 0.039 |
| Superior_corona_radiata_L | External_capsule_L | 0.727 | 0.011 |
| Superior_corona_radiata_L | Superior_longitudinal_fasciculus_R | 0.836 | 0.001 |
| Superior_corona_radiata_L | Superior_longitudinal_fasciculus_L | 0.736 | 0.010 |
| Superior_corona_radiata_L | Superior_fronto_occipital_fasciculus_R | 0.691 | 0.019 |
| Posterior_corona_radiata_R | Anterior_limb_of_internal_capsule_R | 0.736 | 0.010 |
| Posterior_corona_radiata_R | Retrolenticular_part_of_internal_capsule_R | 0.709 | 0.015 |
| Posterior_corona_radiata_R | Anterior_corona_radiata_R | 0.691 | 0.019 |
| Posterior_corona_radiata_R | Anterior_corona_radiata_L | 0.891 | 0.000 |
| Posterior_corona_radiata_R | Superior_corona_radiata_R | 0.818 | 0.002 |
| Posterior_corona_radiata_R | Superior_corona_radiata_L | 0.873 | 0.000 |
| Posterior_corona_radiata_R | External_capsule_R | 0.664 | 0.026 |
| Posterior_corona_radiata_R | External_capsule_L | 0.773 | 0.005 |
| Posterior_corona_radiata_R | Superior_longitudinal_fasciculus_R | 0.827 | 0.002 |
| Posterior_corona_radiata_R | Superior_longitudinal_fasciculus_L | 0.809 | 0.003 |
| Posterior_corona_radiata_L | Anterior_limb_of_internal_capsule_R | 0.664 | 0.026 |
| Posterior_corona_radiata_L | Anterior_limb_of_internal_capsule_L | 0.764 | 0.006 |
| Posterior_corona_radiata_L | Posterior_limb_of_internal_capsule_L | 0.636 | 0.035 |
| Posterior_corona_radiata_L | Retrolenticular_part_of_internal_capsule_R | 0.818 | 0.002 |
| Posterior_corona_radiata_L | Retrolenticular_part_of_internal_capsule_L | 0.627 | 0.039 |
| Posterior_corona_radiata_L | Anterior_corona_radiata_R | 0.882 | 0.000 |
| Posterior_corona_radiata_L | Anterior_corona_radiata_L | 0.782 | 0.004 |
| Posterior_corona_radiata_L | Superior_corona_radiata_R | 0.636 | 0.035 |
| Posterior_corona_radiata_L | Superior_corona_radiata_L | 0.773 | 0.005 |
| Posterior_corona_radiata_L | Sagittal_stratum_L | 0.682 | 0.021 |
| Posterior_corona_radiata_L | External_capsule_R | 0.618 | 0.043 |
| Posterior_corona_radiata_L | External_capsule_L | 0.745 | 0.008 |
| Posterior_corona_radiata_L | Superior_longitudinal_fasciculus_R | 0.682 | 0.021 |
| Posterior_corona_radiata_L | Superior_longitudinal_fasciculus_L | 0.909 | 0.000 |
| Posterior_thalamic_radiation_R | Retrolenticular_part_of_internal_capsule_R | 0.618 | 0.043 |
| Posterior_thalamic_radiation_R | Anterior_corona_radiata_L | 0.627 | 0.039 |
| Posterior_thalamic_radiation_R | Superior_corona_radiata_R | 0.664 | 0.026 |
| Posterior_thalamic_radiation_R | Superior_corona_radiata_L | 0.718 | 0.013 |
| Posterior_thalamic_radiation_R | Superior_fronto_occipital_fasciculus_L | 0.791 | 0.004 |
| Posterior_thalamic_radiation_L | Superior_cerebellar_peduncle_L | -0.645 | 0.032 |
| Posterior_thalamic_radiation_L | Anterior_limb_of_internal_capsule_L | 0.773 | 0.005 |
| Posterior_thalamic_radiation_L | Sagittal_stratum_L | 0.764 | 0.006 |
| Posterior_thalamic_radiation_L | Superior_fronto_occipital_fasciculus_R | 0.627 | 0.039 |
| Posterior_thalamic_radiation_L | Superior_fronto_occipital_fasciculus_L | 0.736 | 0.010 |
| Sagittal_stratum_R | Anterior_corona_radiata_R | 0.700 | 0.016 |
| Sagittal_stratum_R | Anterior_corona_radiata_L | 0.664 | 0.026 |
| Sagittal_stratum_R | External_capsule_R | 0.800 | 0.003 |
| Sagittal_stratum_R | Superior_longitudinal_fasciculus_R | 0.645 | 0.032 |
| Sagittal_stratum_R | Superior_longitudinal_fasciculus_L | 0.764 | 0.006 |
| Sagittal_stratum_L | Anterior_limb_of_internal_capsule_L | 0.682 | 0.021 |
| Sagittal_stratum_L | Retrolenticular_part_of_internal_capsule_R | 0.645 | 0.032 |
| Sagittal_stratum_L | Anterior_corona_radiata_R | 0.618 | 0.043 |
| Sagittal_stratum_L | Superior_fronto_occipital_fasciculus_L | 0.736 | 0.010 |
| External_capsule_R | External_capsule_L | 0.645 | 0.032 |
| External_capsule_R | Superior_longitudinal_fasciculus_R | 0.682 | 0.021 |
| External_capsule_R | Superior_longitudinal_fasciculus_L | 0.782 | 0.004 |
| External_capsule_L | Superior_longitudinal_fasciculus_L | 0.700 | 0.016 |
| Cingulum_R | Superior_cerebellar_peduncle_L | 0.745 | 0.008 |
| Cingulum_R | External_capsule_R | 0.782 | 0.004 |
| Cingulum_R | Superior_longitudinal_fasciculus_L | 0.691 | 0.019 |
| Cingulum_L | Anterior_corona_radiata_L | 0.709 | 0.015 |
| Cingulum_L | External_capsule_R | 0.855 | 0.001 |
| Cingulum_L | Superior_longitudinal_fasciculus_R | 0.673 | 0.023 |
| Fornix_cres_Stria_terminalis_R | Superior_cerebellar_peduncle_L | -0.809 | 0.003 |
| Fornix_cres_Stria_terminalis_L | Superior_corona_radiata_L | 0.627 | 0.039 |
| Fornix_cres_Stria_terminalis_L | Superior_fronto_occipital_fasciculus_R | 0.918 | 0.000 |
| Superior_longitudinal_fasciculus_R | Superior_longitudinal_fasciculus_L | 0.827 | 0.002 |
| Superior_fronto_occipital_fasciculus_R | Superior_fronto_occipital_fasciculus_L | 0.609 | 0.047 |
| Uncinate_fasciculus_R | Posterior_limb_of_internal_capsule_L | 0.664 | 0.026 |
| Uncinate_fasciculus_R | Retrolenticular_part_of_internal_capsule_R | 0.664 | 0.026 |
| Uncinate_fasciculus_R | Retrolenticular_part_of_internal_capsule_L | 0.691 | 0.019 |
| Uncinate_fasciculus_R | External_capsule_R | -0.609 | 0.047 |
| Uncinate_fasciculus_R | Superior_longitudinal_fasciculus_R | 0.627 | 0.039 |
| Uncinate_fasciculus_L | Anterior_limb_of_internal_capsule_R | 0.836 | 0.001 |
| Uncinate_fasciculus_L | Posterior_limb_of_internal_capsule_R | 0.745 | 0.008 |
| Uncinate_fasciculus_L | Retrolenticular_part_of_internal_capsule_R | 0.736 | 0.010 |
| Uncinate_fasciculus_L | Retrolenticular_part_of_internal_capsule_L | 0.736 | 0.010 |
| Uncinate_fasciculus_L | Anterior_corona_radiata_R | 0.627 | 0.039 |
| Uncinate_fasciculus_L | Anterior_corona_radiata_L | 0.845 | 0.001 |
| Uncinate_fasciculus_L | External_capsule_R | 0.809 | 0.003 |
| Uncinate_fasciculus_L | External_capsule_L | 0.682 | 0.021 |
| Uncinate_fasciculus_L | Superior_longitudinal_fasciculus_L | 0.682 | 0.021 |
| Tapetum_R | Retrolenticular_part_of_internal_capsule_L | -0.655 | 0.029 |
| Tapetum_R | Anterior_corona_radiata_R | -0.645 | 0.032 |
| Tapetum_R | Superior_longitudinal_fasciculus_L | -0.682 | 0.021 |
| Tapetum_L | Superior_cerebellar_peduncle_L | -0.664 | 0.026 |

| Supplementary Table S8. Significant correlations of FA metrics between pairs of regions | | | |
| --- | --- | --- | --- |
| Correlation Between | | **Correlation Coefficient (R)** | **P-value** |
| Middle_cerebellar_peduncle | Pontine_crossing_tract | 0.672 | 0.023 |
| Middle_cerebellar_peduncle | Corticospinal_tract_L | 0.681 | 0.021 |
| Pontine_crossing_tract | Middle_cerebellar_peduncle | 0.672 | 0.023 |
| Pontine_crossing_tract | Corticospinal_tract_L | 0.873 | 0.000 |
| Genu_of_corpus_callosum | Body_of_corpus_callosum | 0.695 | 0.018 |
| Genu_of_corpus_callosum | Retrolenticular_part_of_internal_capsule_R | 0.751 | 0.008 |
| Genu_of_corpus_callosum | Retrolenticular_part_of_internal_capsule_L | 0.706 | 0.015 |
| Genu_of_corpus_callosum | Anterior_corona_radiata_R | 0.854 | 0.001 |
| Genu_of_corpus_callosum | Anterior_corona_radiata_L | 0.834 | 0.001 |
| Genu_of_corpus_callosum | Superior_corona_radiata_L | 0.749 | 0.008 |
| Genu_of_corpus_callosum | Posterior_corona_radiata_R | 0.900 | 0.000 |
| Genu_of_corpus_callosum | Sagittal_stratum_L | 0.746 | 0.008 |
| Genu_of_corpus_callosum | Superior_longitudinal_fasciculus_R | 0.716 | 0.013 |
| Genu_of_corpus_callosum | Superior_longitudinal_fasciculus_L | 0.812 | 0.002 |
| Genu_of_corpus_callosum | Uncinate_fasciculus_R | 0.832 | 0.001 |
| Genu_of_corpus_callosum | Uncinate_fasciculus_L | 0.796 | 0.003 |
| Body_of_corpus_callosum | Retrolenticular_part_of_internal_capsule_R | 0.604 | 0.049 |
| Body_of_corpus_callosum | Posterior_corona_radiata_L | 0.617 | 0.043 |
| Body_of_corpus_callosum | Sagittal_stratum_L | 0.746 | 0.008 |
| Body_of_corpus_callosum | Cingulum_R | 0.616 | 0.044 |
| Body_of_corpus_callosum | Superior_longitudinal_fasciculus_R | 0.637 | 0.035 |
| Splenium_of_corpus_callosum | Posterior_corona_radiata_L | 0.608 | 0.047 |
| Splenium_of_corpus_callosum | Posterior_thalamic_radiation_L | 0.749 | 0.008 |
| Splenium_of_corpus_callosum | Cingulum_R | 0.755 | 0.007 |
| Splenium_of_corpus_callosum | Fornix_cres_Stria_terminalis_R | -0.613 | 0.045 |
| Corticospinal_tract_R | Corticospinal_tract_L | 0.826 | 0.002 |
| Corticospinal_tract_L | Middle_cerebellar_peduncle | 0.681 | 0.021 |
| Corticospinal_tract_L | Pontine_crossing_tract | 0.873 | 0.000 |
| Medial_lemniscus_R | Medial_lemniscus_L | 0.777 | 0.005 |
| Medial_lemniscus_L | Cingulum_L | 0.627 | 0.039 |
| Inferior_cerebellar_peduncle_R | Posterior_thalamic_radiation_L | -0.609 | 0.047 |
| Inferior_cerebellar_peduncle_L | Posterior_thalamic_radiation_L | -0.653 | 0.030 |
| Inferior_cerebellar_peduncle_R | Fornix_cres_Stria_terminalis_L | -0.657 | 0.028 |
| Superior_cerebellar_peduncle_R | Superior_cerebellar_peduncle_L | 0.941 | 0.000 |
| Superior_cerebellar_peduncle_R | Tapetum_L | -0.635 | 0.036 |
| Cerebral_peduncle_R | Cerebral_peduncle_L | 0.949 | 0.000 |
| Cerebral_peduncle_R | Retrolenticular_part_of_internal_capsule_R | 0.660 | 0.027 |
| Cerebral_peduncle_R | Retrolenticular_part_of_internal_capsule_L | 0.707 | 0.015 |
| Cerebral_peduncle_R | Cingulum_R | 0.726 | 0.011 |
| Cerebral_peduncle_L | Retrolenticular_part_of_internal_capsule_R | 0.648 | 0.031 |
| Cerebral_peduncle_L | Retrolenticular_part_of_internal_capsule_L | 0.678 | 0.022 |
| Cerebral_peduncle_L | Cingulum_R | 0.615 | 0.044 |
| Retrolenticular_part_of_internal_capsule_L | Retrolenticular_part_of_internal_capsule_R | 0.948 | 0.000 |
| Retrolenticular_part_of_internal_capsule_L | Anterior_corona_radiata_R | 0.865 | 0.001 |
| Retrolenticular_part_of_internal_capsule_L | Anterior_corona_radiata_L | 0.828 | 0.002 |
| Retrolenticular_part_of_internal_capsule_L | Superior_corona_radiata_L | 0.728 | 0.011 |
| Retrolenticular_part_of_internal_capsule_L | Posterior_corona_radiata_R | 0.661 | 0.027 |
| Retrolenticular_part_of_internal_capsule_L | Cingulum_R | 0.604 | 0.049 |
| Retrolenticular_part_of_internal_capsule_L | Superior_longitudinal_fasciculus_L | 0.862 | 0.001 |
| Retrolenticular_part_of_internal_capsule_L | Uncinate_fasciculus_L | 0.691 | 0.018 |
| Retrolenticular_part_of_internal_capsule_L | Tapetum_R | -0.653 | 0.029 |
| Anterior_corona_radiata_R | Retrolenticular_part_of_internal_capsule_R | 0.935 | 0.000 |
| Anterior_corona_radiata_R | Anterior_corona_radiata_L | 0.969 | 0.000 |
| Anterior_corona_radiata_R | Superior_corona_radiata_L | 0.860 | 0.001 |
| Anterior_corona_radiata_R | Posterior_corona_radiata_R | 0.889 | 0.000 |
| Anterior_corona_radiata_R | Posterior_corona_radiata_L | 0.697 | 0.017 |
| Anterior_corona_radiata_R | Superior_longitudinal_fasciculus_R | 0.744 | 0.009 |
| Anterior_corona_radiata_R | Superior_longitudinal_fasciculus_L | 0.894 | 0.000 |
| Anterior_corona_radiata_R | Uncinate_fasciculus_R | 0.631 | 0.037 |
| Anterior_corona_radiata_R | Uncinate_fasciculus_L | 0.857 | 0.001 |
| Anterior_corona_radiata_L | Retrolenticular_part_of_internal_capsule_L | 0.828 | 0.002 |
| Anterior_corona_radiata_L | Superior_corona_radiata_L | 0.881 | 0.000 |
| Anterior_corona_radiata_L | Posterior_corona_radiata_R | 0.893 | 0.000 |
| Anterior_corona_radiata_L | Posterior_corona_radiata_L | 0.639 | 0.034 |
| Anterior_corona_radiata_L | Superior_longitudinal_fasciculus_R | 0.700 | 0.016 |
| Anterior_corona_radiata_L | Superior_longitudinal_fasciculus_L | 0.872 | 0.000 |
| Anterior_corona_radiata_L | Uncinate_fasciculus_R | 0.648 | 0.031 |
| Anterior_corona_radiata_L | Uncinate_fasciculus_L | 0.810 | 0.003 |
| Superior_corona_radiata_L | Retrolenticular_part_of_internal_capsule_R | 0.833 | 0.001 |
| Superior_corona_radiata_L | Posterior_corona_radiata_R | 0.911 | 0.000 |
| Superior_corona_radiata_L | Posterior_corona_radiata_L | 0.791 | 0.004 |
| Superior_corona_radiata_L | Posterior_thalamic_radiation_R | 0.728 | 0.011 |
| Superior_corona_radiata_L | Posterior_thalamic_radiation_L | 0.632 | 0.037 |
| Superior_corona_radiata_L | Fornix_cres_Stria_terminalis_L | 0.653 | 0.029 |
| Superior_corona_radiata_L | Superior_longitudinal_fasciculus_R | 0.736 | 0.010 |
| Superior_corona_radiata_L | Superior_longitudinal_fasciculus_L | 0.863 | 0.001 |
| Superior_corona_radiata_L | Uncinate_fasciculus_L | 0.746 | 0.008 |
| Posterior_corona_radiata_R | Retrolenticular_part_of_internal_capsule_R | 0.748 | 0.004 |
| Posterior_corona_radiata_R | Posterior_corona_radiata_L | 0.758 | 0.007 |
| Posterior_corona_radiata_R | Posterior_thalamic_radiation_R | 0.686 | 0.020 |
| Posterior_corona_radiata_R | Sagittal_stratum_L | 0.647 | 0.031 |
| Posterior_corona_radiata_R | Fornix_cres_Stria_terminalis_L | 0.609 | 0.047 |
| Posterior_corona_radiata_R | Superior_longitudinal_fasciculus_R | 0.768 | 0.006 |
| Posterior_corona_radiata_R | Superior_longitudinal_fasciculus_L | 0.843 | 0.001 |
| Posterior_corona_radiata_R | Uncinate_fasciculus_R | 0.622 | 0.041 |
| Posterior_corona_radiata_R | Uncinate_fasciculus_L | 0.761 | 0.007 |
| Posterior_corona_radiata_L | Retrolenticular_part_of_internal_capsule_R | 0.723 | 0.012 |
| Posterior_corona_radiata_L | Posterior_thalamic_radiation_R | 0.893 | 0.000 |
| Posterior_corona_radiata_L | Posterior_thalamic_radiation_L | 0.835 | 0.001 |
| Posterior_corona_radiata_L | Sagittal_stratum_L | 0.738 | 0.009 |
| Posterior_corona_radiata_L | Superior_longitudinal_fasciculus_R | 0.879 | 0.000 |
| Posterior_corona_radiata_L | Superior_longitudinal_fasciculus_L | 0.786 | 0.004 |
| Posterior_thalamic_radiation_R | Posterior_thalamic_radiation_L | 0.790 | 0.004 |
| Posterior_thalamic_radiation_R | Sagittal_stratum_L | 0.721 | 0.012 |
| Posterior_thalamic_radiation_R | Superior_longitudinal_fasciculus_R | 0.687 | 0.020 |
| Posterior_thalamic_radiation_R | Superior_longitudinal_fasciculus_L | 0.663 | 0.026 |
| Posterior_thalamic_radiation_L | Sagittal_stratum_L | 0.641 | 0.034 |
| Posterior_thalamic_radiation_L | Superior_longitudinal_fasciculus_R | 0.720 | 0.012 |
| Sagittal_stratum_R | Cingulum_L | 0.748 | 0.008 |
| Sagittal_stratum_L | Cingulum_R | 0.723 | 0.012 |
| Sagittal_stratum_L | Cingulum_L | 0.704 | 0.016 |
| Sagittal_stratum_L | Superior_longitudinal_fasciculus_R | 0.801 | 0.003 |
| Sagittal_stratum_L | Superior_longitudinal_fasciculus_L | 0.673 | 0.023 |
| Sagittal_stratum_L | Uncinate_fasciculus_R | 0.677 | 0.022 |
| Cingulum_R | Retrolenticular_part_of_internal_capsule_R | 0.657 | 0.028 |
| Cingulum_R | Cingulum_L | 0.624 | 0.040 |
| Cingulum_R | Fornix_cres_Stria_terminalis_R | -0.613 | 0.045 |
| Cingulum_R | Superior_longitudinal_fasciculus_L | 0.617 | 0.043 |
| Cingulum_L | Superior_longitudinal_fasciculus_R | 0.648 | 0.031 |
| Cingulum_L | Uncinate_fasciculus_R | 0.771 | 0.005 |
| Hippocampus_R | Hippocampus_L | 0.639 | 0.034 |
| Fornix_cres_Stria_terminalis_L | Superior_longitudinal_fasciculus_R | 0.604 | 0.049 |
| Fornix_cres_Stria_terminalis_L | Superior_longitudinal_fasciculus_L | 0.644 | 0.032 |
| Fornix_cres_Stria_terminalis_L | Uncinate_fasciculus_R | 0.695 | 0.018 |
| Superior_longitudinal_fasciculus_R | Retrolenticular_part_of_internal_capsule_R | 0.740 | 0.009 |
| Superior_longitudinal_fasciculus_R | Superior_longitudinal_fasciculus_L | 0.851 | 0.001 |
| Superior_longitudinal_fasciculus_L | Retrolenticular_part_of_internal_capsule_R | 0.943 | 0.000 |
| Superior_longitudinal_fasciculus_L | Uncinate_fasciculus_R | 0.672 | 0.024 |
| Superior_longitudinal_fasciculus_L | Uncinate_fasciculus_L | 0.666 | 0.025 |
| Uncinate_fasciculus_L | Retrolenticular_part_of_internal_capsule_R | 0.686 | 0.020 |
| Tapetum_R | Retrolenticular_part_of_internal_capsule_R | -0.621 | 0.041 |
| Tapetum_R | Tapetum_L | 0.844 | 0.001 |
| Middle_cerebellar_peduncle | External_capsule_L | -0.636 | 0.035 |
| Body_of_corpus_callosum | Posterior_limb_of_internal_capsule_L | 0.609 | 0.047 |
| Corticospinal_tract_R | External_capsule_L | -0.655 | 0.029 |
| Corticospinal_tract_R | Superior_fronto_occipital_fasciculus_L | -0.709 | 0.015 |
| Corticospinal_tract_L | Superior_fronto_occipital_fasciculus_L | -0.691 | 0.019 |
| Inferior_cerebellar_peduncle_R | Superior_fronto_occipital_fasciculus_R | -0.691 | 0.019 |
| Inferior_cerebellar_peduncle_R | Superior_fronto_occipital_fasciculus_L | -0.645 | 0.032 |
| Superior_cerebellar_peduncle_R | Anterior_limb_of_internal_capsule_R | 0.636 | 0.035 |
| Superior_cerebellar_peduncle_R | Anterior_limb_of_internal_capsule_L | 0.655 | 0.029 |
| Superior_cerebellar_peduncle_R | Posterior_limb_of_internal_capsule_R | 0.655 | 0.029 |
| Superior_cerebellar_peduncle_R | External_capsule_R | 0.727 | 0.011 |
| Superior_cerebellar_peduncle_L | External_capsule_R | 0.809 | 0.003 |
| Cerebral_peduncle_R | External_capsule_R | 0.636 | 0.035 |
| Cerebral_peduncle_L | External_capsule_R | 0.664 | 0.026 |
| Anterior_limb_of_internal_capsule_R | Anterior_limb_of_internal_capsule_L | 0.845 | 0.001 |
| Anterior_limb_of_internal_capsule_L | External_capsule_R | 0.673 | 0.023 |
| Posterior_limb_of_internal_capsule_L | Superior_corona_radiata_R | 0.673 | 0.023 |
| Retrolenticular_part_of_internal_capsule_R | Anterior_limb_of_internal_capsule_R | 0.745 | 0.008 |
| Retrolenticular_part_of_internal_capsule_L | Anterior_limb_of_internal_capsule_R | 0.791 | 0.004 |
| Retrolenticular_part_of_internal_capsule_L | Anterior_limb_of_internal_capsule_L | 0.618 | 0.043 |
| Retrolenticular_part_of_internal_capsule_L | External_capsule_R | 0.618 | 0.043 |
| Anterior_corona_radiata_R | Anterior_limb_of_internal_capsule_R | 0.809 | 0.003 |
| Anterior_corona_radiata_R | Anterior_limb_of_internal_capsule_L | 0.727 | 0.011 |
| Anterior_corona_radiata_R | External_capsule_R | 0.673 | 0.023 |
| Anterior_corona_radiata_R | External_capsule_L | 0.636 | 0.035 |
| Anterior_corona_radiata_L | Anterior_limb_of_internal_capsule_R | 0.682 | 0.021 |
| Anterior_corona_radiata_L | External_capsule_R | 0.736 | 0.010 |
| Superior_corona_radiata_L | Superior_fronto_occipital_fasciculus_R | 0.855 | 0.001 |
| Posterior_corona_radiata_R | Posterior_limb_of_internal_capsule_L | 0.727 | 0.011 |
| Posterior_corona_radiata_L | Posterior_limb_of_internal_capsule_L | 0.764 | 0.006 |
| Posterior_corona_radiata_L | Superior_fronto_occipital_fasciculus_R | 0.673 | 0.023 |
| Posterior_thalamic_radiation_R | Posterior_limb_of_internal_capsule_L | 0.682 | 0.021 |
| Posterior_thalamic_radiation_R | Superior_fronto_occipital_fasciculus_R | 0.782 | 0.004 |
| Posterior_thalamic_radiation_L | Superior_fronto_occipital_fasciculus_R | 0.709 | 0.015 |
| Sagittal_stratum_R | External_capsule_R | 0.745 | 0.008 |
| Sagittal_stratum_L | Posterior_limb_of_internal_capsule_L | 0.873 | 0.000 |
| Cingulum_R | External_capsule_R | 0.664 | 0.026 |
| Cingulum_L | External_capsule_R | 0.700 | 0.016 |
| Hippocampus_L | Superior_fronto_occipital_fasciculus_L | -0.700 | 0.016 |
| Fornix_cres_Stria_terminalis_L | Superior_fronto_occipital_fasciculus_R | 0.609 | 0.047 |
| Superior_longitudinal_fasciculus_R | Superior_fronto_occipital_fasciculus_R | 0.618 | 0.043 |
| Superior_longitudinal_fasciculus_L | Posterior_limb_of_internal_capsule_L | 0.664 | 0.026 |
| Uncinate_fasciculus_R | External_capsule_R | 0.627 | 0.039 |
| Uncinate_fasciculus_L | Anterior_limb_of_internal_capsule_R | 0.827 | 0.002 |
| Uncinate_fasciculus_L | Anterior_limb_of_internal_capsule_L | 0.745 | 0.008 |
| Uncinate_fasciculus_L | Superior_fronto_occipital_fasciculus_L | 0.655 | 0.029 |
| Tapetum_R | Anterior_limb_of_internal_capsule_R | -0.636 | 0.035 |
| Tapetum_R | External_capsule_L | -0.609 | 0.047 |
| Tapetum_L | Posterior_limb_of_internal_capsule_R | -0.682 | 0.021 |
| External_capsule_R | Superior_longitudinal_fasciculus_R | 0.682 | 0.021 |
| External_capsule_R | Superior_longitudinal_fasciculus_L | 0.782 | 0.004 |
| External_capsule_L | Superior_longitudinal_fasciculus_L | 0.700 | 0.016 |
| Cingulum_R | Superior_cerebellar_peduncle_L | 0.745 | 0.008 |
| Cingulum_R | External_capsule_R | 0.782 | 0.004 |
| Cingulum_R | Superior_longitudinal_fasciculus_L | 0.691 | 0.019 |
| Cingulum_L | Anterior_corona_radiata_L | 0.709 | 0.015 |
| Cingulum_L | External_capsule_R | 0.855 | 0.001 |
| Cingulum_L | Superior_longitudinal_fasciculus_R | 0.673 | 0.023 |
| Fornix_cres_Stria_terminalis_R | Superior_cerebellar_peduncle_L | -0.809 | 0.003 |
| Fornix_cres_Stria_terminalis_L | Superior_corona_radiata_L | 0.627 | 0.039 |
| Fornix_cres_Stria_terminalis_L | Superior_fronto_occipital_fasciculus_R | 0.918 | 0.000 |
| Superior_longitudinal_fasciculus_R | Superior_longitudinal_fasciculus_L | 0.827 | 0.002 |
| Superior_fronto_occipital_fasciculus_R | Superior_fronto_occipital_fasciculus_L | 0.609 | 0.047 |
| Uncinate_fasciculus_R | Posterior_limb_of_internal_capsule_L | 0.664 | 0.026 |
| Uncinate_fasciculus_R | Retrolenticular_part_of_internal_capsule_R | 0.664 | 0.026 |
| Uncinate_fasciculus_R | Retrolenticular_part_of_internal_capsule_L | 0.691 | 0.019 |
| Uncinate_fasciculus_R | External_capsule_R | -0.609 | 0.047 |
| Uncinate_fasciculus_R | Superior_longitudinal_fasciculus_R | 0.627 | 0.039 |
| Uncinate_fasciculus_L | Anterior_limb_of_internal_capsule_R | 0.836 | 0.001 |
| Uncinate_fasciculus_L | Posterior_limb_of_internal_capsule_R | 0.745 | 0.008 |
| Uncinate_fasciculus_L | Retrolenticular_part_of_internal_capsule_R | 0.736 | 0.010 |
| Uncinate_fasciculus_L | Retrolenticular_part_of_internal_capsule_L | 0.736 | 0.010 |
| Uncinate_fasciculus_L | Anterior_corona_radiata_R | 0.627 | 0.039 |
| Uncinate_fasciculus_L | Anterior_corona_radiata_L | 0.845 | 0.001 |
| Uncinate_fasciculus_L | External_capsule_R | 0.809 | 0.003 |
| Uncinate_fasciculus_L | External_capsule_L | 0.682 | 0.021 |
| Uncinate_fasciculus_L | Superior_longitudinal_fasciculus_L | 0.682 | 0.021 |
| Tapetum_R | Retrolenticular_part_of_internal_capsule_L | -0.655 | 0.029 |
| Tapetum_R | Anterior_corona_radiata_R | -0.645 | 0.032 |
| Tapetum_R | Superior_longitudinal_fasciculus_L | -0.682 | 0.021 |
| Tapetum_L | Superior_cerebellar_peduncle_L | -0.664 | 0.026 |
