# Supplementary material for: Diffusion tensor imaging biomarkers and clinical assessments in amyotrophic lateral sclerosis (ALS) patients: an exploratory study
Source: Ann Med Surg (Lond). 2024 Jul 23;86(9):5080–90. doi: 10.1097/MS9.0000000000002332 (PMC11374192; doi:10.1097/MS9.0000000000002332)
Supplement: Supplementary file 3 [file ms9-86-5080-s003.docx]

| Supplementary Table S9. Significant correlations between FA and clinical parameters | | | |
| --- | --- | --- | --- |
| Correlation Between | | **Correlation Coefficient (R)** | **P-value** |
| MUNIX-Right-median | Pontine_crossing_tract | 0.706 | 0.034 |
| MUNIX-Right-median | Corticospinal_tract_L | 0.679 | 0.044 |
| MUNIX-Right-ulnar | Medial_lemniscus_R | -0.718 | 0.019 |
| MUNIX-Right-ulnar | Medial_lemniscus_L | -0.834 | 0.003 |
| MUNIX-Right-ulnar | Cingulum_L | -0.802 | 0.005 |
| MUNIX-Right-tibial | Medial_lemniscus_R | 0.729 | 0.026 |
| MUNIX-Right-tibial | Sagittal_stratum_R | 0.855 | 0.003 |
| MUNIX-Left-ulnar | Genu_of_corpus_callosum | -0.813 | 0.026 |
| MUNIX-Left-ulnar | Sagittal_stratum_L | -0.899 | 0.007 |
| MUNIX-Left-ulnar | Uncinate_fasciculus_R | -0.896 | 0.006 |
| MUNIX-Left-ulnar | Uncinate_fasciculus_L | 0.768 | 0.044 |
| MUNIX-Left- tibial | Anterior_corona_radiata_L | -0.736 | 0.037 |
| MUNIX-Left- tibial | Cingulum_L | -0.840 | 0.009 |
| MUNIX-Left- tibial | Superior_longitudinal_fasciculus_R | -0.748 | 0.033 |
| MUNIX-Left- tibial | Uncinate_fasciculus_R | -0.756 | 0.030 |
| MUSIX-Right-ulnar | Splenium_of_corpus_callosum | -0.732 | 0.025 |
| MUSIX-Right-ulnar | Medial_lemniscus_R | -0.711 | 0.032 |
| MUSIX-Right-ulnar | Superior_cerebellar_peduncle_L | -0.672 | 0.047 |
| MUSIX-Right-ulnar | Cingulum_R | -0.831 | 0.006 |
| MUSIX-Right-ulnar | Cingulum_L | -0.675 | 0.046 |
| MUSIX-Right-ulnar | Fornix_cres_Stria_terminalis_R | 0.729 | 0.026 |
| MUSIX-Left-ulnar | Genu_of_corpus_callosum | 0.894 | 0.007 |
| MUSIX-Left-ulnar | Anterior_corona_radiata_R | 0.776 | 0.040 |
| MUSIX-Left-ulnar | Posterior_corona_radiata_R | 0.858 | 0.013 |
| MUSIX-Left-ulnar | Sagittal_stratum_L | 0.858 | 0.013 |
| MUSIX-Left-ulnar | Uncinate_fasciculus_R | 0.798 | 0.031 |
| MUSIX-Left-ulnar | Uncinate_fasciculus_L | 0.921 | 0.003 |
| ALSFRS-R | Medial_lemniscus_R | -0.648 | 0.031 |
| ALSFRS-R | Medial_lemniscus_L | -0.638 | 0.035 |
| Dynamo-Right | Tapetum_R | -0.747 | 0.021 |
| Dynamo-Right | Tapetum_L | -0.868 | 0.002 |
| Dynamo-Left | Middle_cerebellar_peduncle | -0.731 | 0.039 |
| MUNIX-Left- tibial | External_capsule_R | -0.714 | 0.047 |
| MUSIX-Right-tibial | Genu_of_corpus_callosum | 0.717 | 0.020 |
| MUSIX-Right-tibial | Anterior_corona_radiata_L | 0.699 | 0.024 |
| MUSIX-Right-tibial | Superior_corona_radiata_L | 0.675 | 0.032 |
| MUSIX-Right-tibial | Superior_corona_radiata_R | 0.821 | 0.004 |
| MUSIX-Right-tibial | Fornix_cres_Stria_terminalis_L | 0.717 | 0.020 |
| MUSIX-Right-tibial | Uncinate_fasciculus_R | 0.754 | 0.012 |
| MUSIX-Right-median | Middle_cerebellar_peduncle | 0.667 | 0.050 |
| MUSIX-Left-tibial | Posterior_limb_of_internal_capsule_L | 0.934 | 0.001 |
| MUSIX-Left-tibial | Superior_fronto_occipital_fasciculus_L | -0.778 | 0.023 |
| Dynamo-Right | Posterior_limb_of_internal_capsule_R | 0.717 | 0.030 |
| Dynamo-Left | External_capsule_L | 0.714 | 0.047 |
| Disease-Duration | Pontine_crossing_tract | -0.616 | 0.044 |
| Disease-Duration | Corticospinal_tract_L | -0.658 | 0.028 |

| Supplementary Table S10. Significant correlations between MD and clinical parameters | | | |
| --- | --- | --- | --- |
| Correlation Between | | **Correlation Coefficient (R)** | **P-value** |
| MUNIX-Right-median | Posterior_thalamic_radiation_L | 0.763 | 0.017 |
| MUNIX-Right-ulnar | Posterior_thalamic_radiation_L | -0.635 | 0.049 |
| MUNIX-Right-ulnar | Fornix_cres_Stria_terminalis_R | -0.671 | 0.034 |
| MUNIX-Right-ulnar | Tapetum_L | 0.657 | 0.039 |
| MUNIX-Right-tibial | Posterior_thalamic_radiation_L | 0.813 | 0.008 |
| MUNIX-Left-ulnar | Retrolenticular_part_of_internal_capsule_R | 0.775 | 0.041 |
| MUNIX-Left-tibial | Medial_lemniscus_L | 0.714 | 0.047 |
| MUNIX-Left-tibial | Cingulum_R | 0.747 | 0.033 |
| MUNIX-Left-tibial | Fornix_cres_Stria_terminalis_R | -0.784 | 0.021 |
| MUNIX-Left-tibial | Tapetum_L | -0.709 | 0.049 |
| MUSIX-Right-ulnar | Inferior_cerebellar_peduncle_L | 0.804 | 0.009 |
| MUSIX-Right-ulnar | Fornix_cres_Stria_terminalis_R | -0.708 | 0.033 |
| Dynamo-Right | Pontine_crossing_tract | -0.667 | 0.050 |
| Dynamo-Right | Posterior_thalamic_radiation_L | 0.736 | 0.024 |
| Dynamo-Right | Cingulum_R | -0.764 | 0.016 |
| Dynamo-Right | Cingulum_L | -0.709 | 0.032 |
| Dynamo-Right | Tapetum_L | 0.683 | 0.043 |
| Dynamo-Left | Middle_cerebellar_peduncle | 0.723 | 0.043 |
| MUNIX-Right-ulnar | Superior_cerebellar_peduncle_L | 0.648 | 0.043 |
| MUNIX-Right-tibial | Superior_cerebellar_peduncle_L | -0.756 | 0.018 |
| MUNIX-Left-ulnar | Retrolenticular_part_of_internal_capsule_L | 0.964 | 0.000 |
| MUNIX-Left-ulnar | Anterior_corona_radiata_R | 0.857 | 0.014 |
| MUNIX-Left-ulnar | Anterior_corona_radiata_L | 0.786 | 0.036 |
| MUNIX-Left-tibial | Superior_longitudinal_fasciculus_R | 0.810 | 0.015 |
| MUSIX-Right-tibial | Posterior_corona_radiata_R | -0.687 | 0.028 |
| MUSIX-Right-tibial | Superior_longitudinal_fasciculus_L | -0.644 | 0.044 |
| MUSIX-Right-ulnar | Superior_cerebellar_peduncle_L | 0.767 | 0.016 |
| MUSIX-Right-ulnar | Anterior_corona_radiata_R | 0.767 | 0.016 |
| MUSIX-Right-median | Middle_cerebellar_peduncle | -0.733 | 0.025 |
| MUSIX-Left-ulnar | Superior_cerebellar_peduncle_R | 0.786 | 0.036 |
| ALSFRS-R | Posterior_limb_of_internal_capsule_R | -0.769 | 0.006 |
| ALSFRS-R | Posterior_limb_of_internal_capsule_L | -0.696 | 0.017 |
| Dynamo-Left | Hippocampus_L | 0.714 | 0.047 |
| Disease-Duration | Body_of_corpus_callosum | 0.635 | 0.036 |
| Disease-Duration | Corticospinal_tract_L | 0.851 | 0.001 |
| Disease-Duration | Inferior_cerebellar_peduncle_R | 0.667 | 0.025 |

| Supplementary Table S11. Significant correlations between RD and clinical parameters | | | |
| --- | --- | --- | --- |
| Correlation Between | | **Correlation Coefficient (R)** | **P-value** |
| MUNIX-Right-median | Superior_cerebellar_peduncle_R | 0.755 | 0.019 |
| MUNIX-Right-median | Posterior_thalamic_radiation_L | 0.779 | 0.013 |
| MUNIX-Right-tibial | Posterior_thalamic_radiation_L | 0.759 | 0.018 |
| MUNIX-Left-ulnar | Uncinate_fasciculus_R | 0.833 | 0.020 |
| MUNIX-Left-tibial | Medial_lemniscus_L | 0.716 | 0.046 |
| MUNIX-Left-tibial | Cingulum_L | 0.821 | 0.012 |
| MUNIX-Left-tibial | Fornix_cres_Stria_terminalis_R | 0.711 | 0.048 |
| MUSIX-Right-ulnar | Inferior_cerebellar_peduncle_L | 0.775 | 0.014 |
| MUSIX-Right-ulnar | Cingulum_R | 0.768 | 0.016 |
| MUSIX-Right-ulnar | Fornix_cres_Stria_terminalis_R | -0.777 | 0.014 |
| MUSIX-Left-ulnar | Posterior_corona_radiata_R | -0.789 | 0.035 |
| MUSIX-Left-ulnar | Uncinate_fasciculus_R | -0.819 | 0.024 |
| MUSIX-Left-ulnar | Uncinate_fasciculus_L | -0.845 | 0.017 |
| Dynamo-Right | Posterior_thalamic_radiation_L | 0.755 | 0.019 |
| Dynamo-Right | Tapetum_L | 0.733 | 0.025 |
| Dynamo-Left | Middle_cerebellar_peduncle | 0.738 | 0.037 |
| MUNIX-Right-median | Sagittal_stratum_L | 0.800 | 0.010 |
| MUNIX-Right-median | Superior_fronto_occipital_fasciculus_L | 0.700 | 0.036 |
| MUNIX-Right-tibial | Superior_cerebellar_peduncle_L | -0.740 | 0.023 |
| MUNIX-Left-ulnar | Retrolenticular_part_of_internal_capsule_R | 0.821 | 0.023 |
| MUNIX-Left-ulnar | Retrolenticular_part_of_internal_capsule_L | 0.857 | 0.014 |
| MUNIX-Left-ulnar | Anterior_corona_radiata_R | 0.893 | 0.007 |
| MUNIX-Left-ulnar | Superior_corona_radiata_L | 0.786 | 0.036 |
| MUNIX-Left-ulnar | Superior_longitudinal_fasciculus_R | 0.786 | 0.036 |
| MUNIX-Left-tibial | External_capsule_R | 0.762 | 0.028 |
| MUNIX-Left-tibial | Hippocampus_L | 0.762 | 0.028 |
| MUNIX-Left-tibial | Superior_longitudinal_fasciculus_R | 0.833 | 0.010 |
| MUSIX-Right-tibial | Genu_of_corpus_callosum | -0.705 | 0.023 |
| MUSIX-Right-tibial | Anterior_corona_radiata_L | -0.675 | 0.032 |
| MUSIX-Right-ulnar | Superior_cerebellar_peduncle_L | 0.700 | 0.036 |
| MUSIX-Right-median | Middle_cerebellar_peduncle | -0.733 | 0.025 |
| MUSX-Right-median | Corticospinal_tract_L | -0.667 | 0.050 |
| MUSIX-Left-tibial | Posterior_limb_of_internal_capsule_L | -0.731 | 0.040 |
| Dynamo-Right | Sagittal_stratum_L | 0.767 | 0.016 |
| Disease-Duration | Pontine_crossing_tract | 0.607 | 0.048 |
| Disease-Duration | Corticospinal_tract_L | 0.782 | 0.004 |
| Disease-Duration | Inferior_cerebellar_peduncle_R | 0.621 | 0.042 |

| Supplementary Table S12. Significant correlations between AD and clinical parameters | | | |
| --- | --- | --- | --- |
| Correlation Between | | **Correlation Coefficient (R)** | **P-value** |
| MUNIX-Right-median | Posterior_thalamic_radiation_L | 0.672 | 0.047 |
| MUNIX-Right-median | Fornix_cres_Stria_terminalis_R | 0.741 | 0.022 |
| MUNIX-Right-ulnar | Posterior_thalamic_radiation_L | -0.748 | 0.013 |
| MUNIX-Right-ulnar | Fornix_cres_Stria_terminalis_R | -0.729 | 0.017 |
| MUNIX-Right-ulnar | Tapetum_L | -0.714 | 0.020 |
| MUNIX-Right-tibial | Posterior_thalamic_radiation_L | 0.870 | 0.002 |
| MUNIX-Left-tibial | Fornix | -0.712 | 0.048 |
| MUNIX-Left-tibial | Superior_cerebellar_peduncle_R | -0.801 | 0.017 |
| MUNIX-Left-tibial | Fornix_cres_Stria_terminalis_R | -0.826 | 0.011 |
| MUNIX-Left-tibial | Tapetum_L | -0.742 | 0.035 |
| MUSIX-Right-ulnar | Fornix | -0.767 | 0.016 |
| MUSIX-Right-ulnar | Inferior_cerebellar_peduncle_L | 0.833 | 0.005 |
| MUSIX-Right-ulnar | Superior_cerebellar_peduncle_L | 0.804 | 0.009 |
| MUSIX-Right-ulnar | Cingulum_R | -0.706 | 0.034 |
| MUSIX-Right-ulnar | Uncinate_fasciculus_L | 0.671 | 0.048 |
| MUSIX-Right-ulnar | Tapetum_L | -0.707 | 0.033 |
| MUSIX-Left- tibial | Cingulum_L | 0.812 | 0.014 |
| ALSFRS-R | Cingulum_L | -0.731 | 0.011 |
| Dynamo-Right | Genu_of_corpus_callosum | 0.987 | 0.041 |
| MUNIX-Right-median | Posterior_limb_of_internal_capsule_R | 0.783 | 0.013 |
| MUNIX-Right-median | Posterior_limb_of_internal_capsule_L | 0.750 | 0.020 |
| MUNIX-Right-median | Anterior_corona_radiata_L | 0.800 | 0.010 |
| MUNIX-Right-ulnar | Posterior_limb_of_internal_capsule_L | -0.648 | 0.043 |
| MUNIX-Right-tibial | Posterior_limb_of_internal_capsule_L | 0.740 | 0.023 |
| MUNIX-Left-ulnar | Superior_corona_radiata_R | 0.786 | 0.036 |
| MUNIX-Left-ulnar | External_capsule_L | 0.786 | 0.036 |
| MUNIX-Left-tibial | Superior_longitudinal_fasciculus_L | 0.714 | 0.047 |
| MUSIX-Right-ulnar | Anterior_corona_radiata_R | 0.733 | 0.025 |
| MUSIX-Right-ulnar | Superior_corona_radiata_R | 0.667 | 0.050 |
| MUSIX-Right-ulnar | Superior_corona_radiata_L | 0.800 | 0.010 |
| MUSIX-Right-median | Middle_cerebellar_peduncle | -0.667 | 0.050 |
| MUSIX-Right-median | Anterior_limb_of_internal_capsule_L | 0.750 | 0.020 |
| ALSFRS-R | Superior_longitudinal_fasciculus_R | -0.705 | 0.015 |
| Dynamo-Right | Posterior_limb_of_internal_capsule_R | 0.700 | 0.036 |
| Disease-Duration | Corticospinal_tract_L | 0.847 | 0.000 |
| Disease-Duration | Inferior_cerebellar_peduncle_R | 0.731 | 0.011 |
