# Supplementary material for: Diffusion tensor imaging biomarkers and clinical assessments in amyotrophic lateral sclerosis (ALS) patients: an exploratory study
Source: Ann Med Surg (Lond). 2024 Jul 23;86(9):5080–90. doi: 10.1097/MS9.0000000000002332 (PMC11374192; doi:10.1097/MS9.0000000000002332)
Supplement: Supplementary file 4 [file ms9-86-5080-s004.docx]

| Supplementary Table S1. Comparison of FA metrics between groups | | | | | |
| --- | --- | --- | --- | --- | --- |
| Imaging Parameters | **Cases** | | **Controls** | | **P-value** |
|  | **Mean** | **SD** | **Mean** | **SD** |  |
| Middle_cerebellar_peduncle | 0.3497 | 0.0186 | 0.3622 | 0.0354 | 0.281 |
| Pontine_crossing_tract | 0.2895 | 0.0432 | 0.3234 | 0.0537 | 0.081 |
| Genu_of_corpus_callosum | 0.4765 | 0.0318 | 0.4873 | 0.0293 | 0.344 |
| Body_of_corpus_callosum | 0.5092 | 0.0504 | 0.5230 | 0.0346 | 0.858 |
| Splenium_of_corpus_callosum | 0.5402 | 0.0545 | 0.5526 | 0.0528 | 0.537 |
| Fornix | 0.3040 | 0.0704 | 0.3284 | 0.0873 | 0.430 |
| Corticospinal_tract_R | 0.2571 | 0.0605 | 0.3106 | 0.0767 | 0.054 |
| Corticospinal_tract_L | 0.2698 | 0.0579 | 0.3105 | 0.0683 | 0.103 |
| Medial_lemniscus_R | 0.3756 | 0.0430 | 0.3804 | 0.0575 | 0.810 |
| Medial_lemniscus_L | 0.3857 | 0.0492 | 0.3854 | 0.0499 | 0.990 |
| Inferior_cerebellar_peduncle_R | 0.3125 | 0.0328 | 0.3091 | 0.0392 | 0.810 |
| Inferior_cerebellar_peduncle_L | 0.3015 | 0.0174 | 0.3139 | 0.0392 | 0.326 |
| Superior_cerebellar_peduncle_R | 0.4264 | 0.0910 | 0.4138 | 0.1207 | 0.763 |
| Superior_cerebellar_peduncle_L | 0.4160 | 0.1060 | 0.3993 | 0.1105 | 0.683 |
| Cerebral_peduncle_R | **0.4302** | **0.0522** | **0.4752** | **0.0484** | **0.021** |
| Cerebral_peduncle_L | **0.4341** | **0.0524** | **0.4765** | **0.0418** | **0.018** |
| Anterior_limb_of_internal_capsule_R | 0.4763 | 0.0633 | 0.4833 | 0.0255 | 0.858 |
| Anterior_limb_of_internal_capsule_L | 0.4797 | 0.0584 | 0.5003 | 0.0247 | 0.331 |
| Posterior_limb_of_internal_capsule_R | 0.4628 | 0.0730 | 0.4858 | 0.0342 | 0.394 |
| Posterior_limb_of_internal_capsule_L | 0.4626 | 0.0645 | 0.4910 | 0.0262 | 0.084 |
| Retrolenticular_part_of_internal_capsule_R | 0.4928 | 0.0366 | 0.4881 | 0.0426 | 0.758 |
| Retrolenticular_part_of_internal_capsule_L | 0.4940 | 0.0609 | 0.4955 | 0.0369 | 0.932 |
| Anterior_corona_radiata_R | 0.4194 | 0.0524 | 0.4392 | 0.0302 | 0.183 |
| Anterior_corona_radiata_L | 0.4190 | 0.0742 | 0.4351 | 0.0390 | 0.796 |
| Superior_corona_radiata_R | 0.4266 | 0.0471 | 0.4450 | 0.0499 | 0.065 |
| Superior_corona_radiata_L | 0.4200 | 0.0490 | 0.4339 | 0.0691 | 0.092 |
| Posterior_corona_radiata_R | 0.4145 | 0.0464 | 0.4088 | 0.0357 | 0.700 |
| Posterior_corona_radiata_L | 0.4321 | 0.0568 | 0.4256 | 0.0368 | 0.699 |
| Posterior_thalamic_radiation_R | 0.5023 | 0.0397 | 0.5029 | 0.0436 | 0.970 |
| Posterior_thalamic_radiation_L | 0.4772 | 0.0487 | 0.4800 | 0.0366 | 0.858 |
| Sagittal_stratum_R | 0.4448 | 0.0284 | 0.4405 | 0.0532 | 0.805 |
| Sagittal_stratum_L | 0.4181 | 0.0279 | 0.4199 | 0.0381 | 0.895 |
| External_capsule_R | 0.3423 | 0.0452 | 0.3586 | 0.0408 | 0.071 |
| External_capsule_L | 0.3567 | 0.0555 | 0.3803 | 0.0268 | 0.065 |
| Cingulum_R | 0.4395 | 0.0630 | 0.4010 | 0.0837 | 0.191 |
| Cingulum_L | 0.4051 | 0.0360 | 0.3658 | 0.0722 | 0.101 |
| Hippocampus_R | 0.2603 | 0.0510 | 0.2906 | 0.0269 | 0.038 |
| Hippocampus_L | **0.2443** | **0.0613** | **0.2836** | **0.0409** | **0.041** |
| Fornix_cres_Stria_terminalis_R | 0.3026 | 0.0335 | 0.3021 | 0.0407 | 0.975 |
| Fornix_cres_Stria_terminalis_L | 0.3394 | 0.0258 | 0.3536 | 0.0326 | 0.223 |
| Superior_longitudinal_fasciculus_R | 0.4225 | 0.0419 | 0.4189 | 0.0615 | 0.648 |
| Superior_longitudinal_fasciculus_L | 0.4132 | 0.0428 | 0.4426 | 0.0875 | 0.648 |
| Superior_fronto_occipital_fasciculus_R | 0.3410 | 0.0779 | 0.3324 | 0.0860 | 0.565 |
| Superior_fronto_occipital_fasciculus_L | 0.3542 | 0.0978 | 0.3798 | 0.0715 | 0.921 |
| Uncinate_fasciculus_R | 0.3270 | 0.0608 | 0.3572 | 0.0592 | 0.185 |
| Uncinate_fasciculus_L | 0.4082 | 0.0517 | 0.4150 | 0.0629 | 0.763 |
| Tapetum_R | 0.2318 | 0.0412 | 0.2605 | 0.0777 | 0.265 |
| Tapetum_L | 0.1960 | 0.0626 | 0.2480 | 0.0889 | 0.095 |
| Supplementary Table S2. Comparison of MD metrics between groups | | | | | |
| Imaging Parameters | **Cases** | | **Controls** | | **P-value** |
|  | **Mean** | **SD** | **Mean** | **SD** |  |
| Middle_cerebellar_peduncle | 0.0013 | 0.0002 | 0.0230 | 0.0972 | 0.967 |
| Pontine_crossing_tract | **0.0012** | **0.0002** | **0.0010** | **0.0002** | **0.042** |
| Genu_of_corpus_callosum | 0.0011 | 0.0001 | 0.0447 | 0.1948 | 0.322 |
| Body_of_corpus_callosum | 0.0010 | 0.0001 | 0.0010 | 0.0001 | 0.504 |
| Splenium_of_corpus_callosum | 0.0009 | 0.0001 | 0.0009 | 0.0001 | 0.849 |
| Fornix | 0.0019 | 0.0003 | 0.0018 | 0.0004 | 0.533 |
| Corticospinal_tract_R | 0.0017 | 0.0003 | 0.0216 | 0.0898 | 0.069 |
| Corticospinal_tract_L | 0.0016 | 0.0002 | 0.0016 | 0.0004 | 0.646 |
| Medial_lemniscus_R | 0.0010 | 0.0001 | 0.0010 | 0.0002 | 0.108 |
| Medial_lemniscus_L | 0.0010 | 0.0002 | 0.0011 | 0.0005 | 0.736 |
| Inferior_cerebellar_peduncle_R | 0.0010 | 0.0001 | 0.0299 | 0.1324 | 0.796 |
| Inferior_cerebellar_peduncle_L | 0.0010 | 0.0001 | 0.0010 | 0.0001 | 0.251 |
| Superior_cerebellar_peduncle_R | 0.0012 | 0.0001 | 0.0485 | 0.2116 | 0.680 |
| Superior_cerebellar_peduncle_L | 0.0012 | 0.0002 | 0.0012 | 0.0003 | 0.715 |
| Cerebral_peduncle_R | **0.0012** | **0.0002** | **0.0011** | **0.0001** | **0.045** |
| Cerebral_peduncle_L | 0.0011 | 0.0001 | 0.0011 | 0.0001 | 0.117 |
| Anterior_limb_of_internal_capsule_R | **0.0008** | **0.0001** | **0.0007** | **0.0000** | **0.009** |
| Anterior_limb_of_internal_capsule_L | **0.0008** | **0.0002** | **0.0007** | **0.0000** | **0.015** |
| Posterior_limb_of_internal_capsule_R | **0.0008** | **0.0001** | **0.0007** | **0.0001** | **0.001** |
| Posterior_limb_of_internal_capsule_L | **0.0008** | **0.0002** | **0.0007** | **0.0000** | **0.003** |
| Retrolenticular_part_of_internal_capsule_R | 0.0009 | 0.0001 | 0.0009 | 0.0001 | 0.375 |
| Retrolenticular_part_of_internal_capsule_L | 0.0009 | 0.0001 | 0.0008 | 0.0000 | 0.539 |
| Anterior_corona_radiata_R | 0.0008 | 0.0001 | 0.0014 | 0.0028 | 0.231 |
| Anterior_corona_radiata_L | 0.0008 | 0.0002 | 0.0026 | 0.0083 | 0.148 |
| Superior_corona_radiata_R | 0.0008 | 0.0001 | 0.0007 | 0.0000 | 0.052 |
| Superior_corona_radiata_L | **0.0008** | **0.0001** | **0.0007** | **0.0000** | **0.004** |
| Posterior_corona_radiata_R | 0.0009 | 0.0001 | 0.0046 | 0.0167 | 0.386 |
| Posterior_corona_radiata_L | 0.0009 | 0.0001 | 0.0079 | 0.0314 | 0.201 |
| Posterior_thalamic_radiation_R | 0.0010 | 0.0001 | 0.0093 | 0.0383 | 0.677 |
| Posterior_thalamic_radiation_L | 0.0010 | 0.0001 | 0.0420 | 0.1770 | 0.827 |
| Sagittal_stratum_R | 0.0009 | 0.0000 | 0.0009 | 0.0001 | 0.463 |
| Sagittal_stratum_L | 0.0010 | 0.0001 | 0.0300 | 0.1330 | 0.648 |
| External_capsule_R | **0.0008** | **0.0002** | **0.0139** | **0.0601** | **0.004** |
| External_capsule_L | **0.0008** | **0.0002** | **0.0008** | **0.0000** | **0.009** |
| Cingulum_R | 0.0008 | 0.0000 | 0.0008 | 0.0000 | 0.101 |
| Cingulum_L | 0.0008 | 0.0000 | 0.0093 | 0.0393 | 0.592 |
| Hippocampus_R | 0.0011 | 0.0002 | 0.0217 | 0.0950 | 0.197 |
| Hippocampus_L | **0.0012** | **0.0003** | **0.0010** | **0.0001** | **0.003** |
| Fornix_cres_Stria_terminalis_R | 0.0011 | 0.0001 | 0.0679 | 0.2176 | 0.539 |
| Fornix_cres_Stria_terminalis_L | **0.0012** | **0.0001** | **0.0011** | **0.0001** | **0.030** |
| Superior_longitudinal_fasciculus_R | 0.0008 | 0.0001 | 0.0009 | 0.0006 | 0.901 |
| Superior_longitudinal_fasciculus_L | 0.0008 | 0.0001 | 0.0007 | 0.0000 | 0.201 |
| Superior_fronto_occipital_fasciculus_R | 0.0008 | 0.0002 | 0.0199 | 0.0879 | 0.984 |
| Superior_fronto_occipital_fasciculus_L | 0.0008 | 0.0003 | 0.0007 | 0.0001 | 0.083 |
| Uncinate_fasciculus_R | 0.0008 | 0.0001 | 0.0008 | 0.0001 | 0.160 |
| Uncinate_fasciculus_L | **0.0008** | **0.0000** | **0.0009** | **0.0006** | **0.037** |
| Tapetum_R | 0.0022 | 0.0003 | 0.0020 | 0.0004 | 0.336 |
| Tapetum_L | 0.0023 | 0.0004 | 0.0020 | 0.0004 | 0.052 |
| Supplementary Table S3. Comparison of RD metrics between groups | | | | | |
| Imaging Parameters | **Cases** | | **Controls** | | **P-value** |
|  | **Mean** | **SD** | **Mean** | **SD** |  |
| Middle_cerebellar_peduncle | 0.0011 | 0.0002 | 0.0010 | 0.0002 | 0.798 |
| Pontine_crossing_tract | 0.0010 | 0.0002 | 0.0145 | 0.0623 | 0.092 |
| Genu_of_corpus_callosum | 0.0008 | 0.0001 | 0.0009 | 0.0001 | 0.437 |
| Body_of_corpus_callosum | 0.0007 | 0.0001 | 0.0007 | 0.0001 | 0.309 |
| Splenium_of_corpus_callosum | 0.0006 | 0.0001 | 0.0006 | 0.0001 | 0.759 |
| Fornix | 0.0016 | 0.0003 | 0.0756 | 0.3313 | 0.620 |
| Corticospinal_tract_R | 0.0016 | 0.0003 | 0.0013 | 0.0004 | 0.149 |
| Corticospinal_tract_L | 0.0015 | 0.0002 | 0.0014 | 0.0004 | 0.575 |
| Medial_lemniscus_R | 0.0008 | 0.0001 | 0.0068 | 0.0278 | 0.275 |
| Medial_lemniscus_L | 0.0008 | 0.0001 | 0.0007 | 0.0001 | 0.649 |
| Inferior_cerebellar_peduncle_R | 0.0009 | 0.0001 | 0.0577 | 0.1796 | 0.827 |
| Inferior_cerebellar_peduncle_L | 0.0008 | 0.0001 | 0.0008 | 0.0001 | 0.315 |
| Superior_cerebellar_peduncle_R | 0.0009 | 0.0002 | 0.0482 | 0.2116 | 0.650 |
| Superior_cerebellar_peduncle_L | 0.0010 | 0.0003 | 0.0010 | 0.0003 | 0.715 |
| Cerebral_peduncle_R | **0.0009** | **0.0002** | **0.0008** | **0.0001** | **0.024** |
| Cerebral_peduncle_L | 0.0009 | 0.0001 | 0.0008 | 0.0001 | 0.075 |
| Anterior_limb_of_internal_capsule_R | 0.0005 | 0.0001 | 0.0088 | 0.0382 | 0.197 |
| Anterior_limb_of_internal_capsule_L | 0.0005 | 0.0002 | 0.0391 | 0.1771 | 0.050 |
| Posterior_limb_of_internal_capsule_R | **0.0006** | **0.0001** | **0.0005** | **0.0000** | **0.028** |
| Posterior_limb_of_internal_capsule_L | **0.0006** | **0.0002** | **0.0295** | **0.1330** | **0.012** |
| Retrolenticular_part_of_internal_capsule_R | 0.0006 | 0.0001 | 0.0137 | 0.0600 | 0.796 |
| Retrolenticular_part_of_internal_capsule_L | 0.0006 | 0.0001 | 0.0006 | 0.0000 | 0.680 |
| Anterior_corona_radiata_R | 0.0006 | 0.0001 | 0.0012 | 0.0028 | 0.215 |
| Anterior_corona_radiata_L | 0.0006 | 0.0002 | 0.0109 | 0.0396 | 0.463 |
| Superior_corona_radiata_R | 0.0006 | 0.0001 | 0.0213 | 0.0950 | 0.050 |
| Superior_corona_radiata_L | **0.0006** | **0.0001** | **0.0005** | **0.0000** | **0.008** |
| Posterior_corona_radiata_R | 0.0007 | 0.0001 | 0.0457 | 0.1900 | 0.858 |
| Posterior_corona_radiata_L | 0.0007 | 0.0001 | 0.0077 | 0.0314 | 0.364 |
| Posterior_thalamic_radiation_R | 0.0007 | 0.0001 | 0.0007 | 0.0001 | 0.939 |
| Posterior_thalamic_radiation_L | 0.0007 | 0.0001 | 0.0031 | 0.0110 | 0.483 |
| Sagittal_stratum_R | 0.0007 | 0.0000 | 0.0198 | 0.0878 | 0.592 |
| Sagittal_stratum_L | 0.0007 | 0.0001 | 0.0007 | 0.0001 | 0.342 |
| External_capsule_R | **0.0007** | **0.0002** | **0.0006** | **0.0000** | **0.002** |
| External_capsule_L | **0.0007** | **0.0002** | **0.0007** | **0.0006** | **0.023** |
| Cingulum_R | 0.0006 | 0.0000 | 0.0006 | 0.0000 | 0.819 |
| Cingulum_L | 0.0006 | 0.0000 | 0.0006 | 0.0000 | 0.481 |
| Hippocampus_R | 0.0009 | 0.0002 | 0.0009 | 0.0002 | 0.107 |
| Hippocampus_L | **0.0011** | **0.0003** | **0.0008** | **0.0002** | **0.003** |
| Fornix_cres_Stria_terminalis_R | 0.0010 | 0.0001 | 0.0262 | 0.1157 | 0.312 |
| Fornix_cres_Stria_terminalis_L | **0.0010** | **0.0001** | **0.0009** | **0.0001** | **0.048** |
| Superior_longitudinal_fasciculus_R | 0.0006 | 0.0001 | 0.0007 | 0.0006 | 0.796 |
| Superior_longitudinal_fasciculus_L | 0.0006 | 0.0001 | 0.0006 | 0.0000 | 0.463 |
| Superior_fronto_occipital_fasciculus_R | 0.0007 | 0.0002 | 0.0006 | 0.0001 | 0.984 |
| Superior_fronto_occipital_fasciculus_L | 0.0007 | 0.0003 | 0.0006 | 0.0001 | 0.186 |
| Uncinate_fasciculus_R | 0.0007 | 0.0001 | 0.0006 | 0.0001 | 0.127 |
| Uncinate_fasciculus_L | 0.0006 | 0.0000 | 0.0006 | 0.0001 | 0.258 |
| Tapetum_R | 0.0019 | 0.0003 | 0.0018 | 0.0004 | 0.451 |
| Tapetum_L | 0.0021 | 0.0004 | 0.0018 | 0.0004 | 0.067 |
| Supplementary Table S4. Comparison of AD metrics between groups | | | | | |
| Imaging Parameters | **Cases** | | **Controls** | | **P-value** |
|  | **Mean** | **SD** | **Mean** | **SD** |  |
| Middle_cerebellar_peduncle | 0.0016 | 0.0002 | 0.0016 | 0.0002 | 0.956 |
| Pontine_crossing_tract | 0.0015 | 0.0002 | 0.0014 | 0.0002 | 0.063 |
| Genu_of_corpus_callosum | 0.0017 | 0.0001 | 0.0017 | 0.0001 | 0.196 |
| Body_of_corpus_callosum | 0.0016 | 0.0002 | 0.0016 | 0.0002 | 0.748 |
| Splenium_of_corpus_callosum | 0.0015 | 0.0002 | 0.0016 | 0.0002 | 0.850 |
| Fornix | 0.0025 | 0.0003 | 0.0024 | 0.0004 | 0.340 |
| Corticospinal_tract_R | 0.0021 | 0.0002 | 0.0019 | 0.0004 | 0.116 |
| Corticospinal_tract_L | 0.0020 | 0.0002 | 0.0020 | 0.0003 | 0.819 |
| Medial_lemniscus_R | 0.0014 | 0.0001 | 0.0014 | 0.0003 | 0.231 |
| Medial_lemniscus_L | 0.0014 | 0.0002 | 0.0013 | 0.0002 | 0.514 |
| Inferior_cerebellar_peduncle_R | 0.0014 | 0.0001 | 0.0317 | 0.1357 | 0.804 |
| Inferior_cerebellar_peduncle_L | 0.0013 | 0.0001 | 0.0013 | 0.0001 | 0.415 |
| Superior_cerebellar_peduncle_R | 0.0017 | 0.0002 | 0.0490 | 0.2117 | 0.934 |
| Superior_cerebellar_peduncle_L | 0.0018 | 0.0002 | 0.0017 | 0.0003 | 0.637 |
| Cerebral_peduncle_R | 0.0017 | 0.0001 | 0.0016 | 0.0001 | 0.079 |
| Cerebral_peduncle_L | 0.0016 | 0.0001 | 0.0016 | 0.0001 | 0.429 |
| Anterior_limb_of_internal_capsule_R | 0.0012 | 0.0001 | 0.0095 | 0.0381 | 0.062 |
| Anterior_limb_of_internal_capsule_L | 0.0012 | 0.0002 | 0.0398 | 0.1770 | 0.331 |
| Posterior_limb_of_internal_capsule_R | 0.0012 | 0.0001 | 0.0011 | 0.0001 | 0.092 |
| Posterior_limb_of_internal_capsule_L | 0.0012 | 0.0001 | 0.0301 | 0.1328 | 0.331 |
| Retrolenticular_part_of_internal_capsule_R | 0.0014 | 0.0001 | 0.0144 | 0.0599 | 0.796 |
| Retrolenticular_part_of_internal_capsule_L | 0.0013 | 0.0001 | 0.0013 | 0.0000 | 0.158 |
| Anterior_corona_radiata_R | 0.0011 | 0.0001 | 0.0017 | 0.0028 | 0.265 |
| Anterior_corona_radiata_L | 0.0012 | 0.0002 | 0.0114 | 0.0395 | 0.148 |
| Superior_corona_radiata_R | 0.0011 | 0.0001 | 0.0218 | 0.0949 | 0.463 |
| Superior_corona_radiata_L | 0.0011 | 0.0001 | 0.0011 | 0.0000 | 0.127 |
| Posterior_corona_radiata_R | 0.0014 | 0.0001 | 0.0463 | 0.1899 | 0.171 |
| Posterior_corona_radiata_L | **0.0013** | **0.0000** | **0.0083** | **0.0314** | **0.010** |
| Posterior_thalamic_radiation_R | 0.0015 | 0.0001 | 0.0015 | 0.0001 | 0.662 |
| Posterior_thalamic_radiation_L | 0.0016 | 0.0001 | 0.0040 | 0.0109 | 0.620 |
| Sagittal_stratum_R | 0.0014 | 0.0001 | 0.0205 | 0.0876 | 0.766 |
| Sagittal_stratum_L | 0.0014 | 0.0001 | 0.0014 | 0.0001 | 0.563 |
| External_capsule_R | **0.0012** | **0.0002** | **0.0011** | **0.0001** | **0.010** |
| External_capsule_L | 0.0012 | 0.0002 | 0.0012 | 0.0005 | 0.197 |
| Cingulum_R | **0.0012** | **0.0001** | **0.0011** | **0.0001** | **0.037** |
| Cingulum_L | 0.0011 | 0.0000 | 0.0011 | 0.0001 | 0.050 |
| Hippocampus_R | 0.0014 | 0.0002 | 0.0013 | 0.0002 | 0.283 |
| Hippocampus_L | **0.0015** | **0.0003** | **0.0013** | **0.0001** | **0.009** |
| Fornix_cres_Stria_terminalis_R | 0.0015 | 0.0001 | 0.0267 | 0.1157 | 0.463 |
| Fornix_cres_Stria_terminalis_L | **0.0016** | **0.0001** | **0.0014** | **0.0002** | **0.048** |
| Superior_longitudinal_fasciculus_R | 0.0011 | 0.0001 | 0.0012 | 0.0006 | 0.766 |
| Superior_longitudinal_fasciculus_L | 0.0011 | 0.0001 | 0.0011 | 0.0001 | 0.159 |
| Superior_fronto_occipital_fasciculus_R | 0.0011 | 0.0002 | 0.0011 | 0.0001 | 0.487 |
| Superior_fronto_occipital_fasciculus_L | 0.0011 | 0.0002 | 0.0010 | 0.0001 | 0.026 |
| Uncinate_fasciculus_R | 0.0011 | 0.0001 | 0.0011 | 0.0001 | 0.649 |
| Uncinate_fasciculus_L | 0.0012 | 0.0001 | 0.0011 | 0.0001 | 0.312 |
| Tapetum_R | 0.0026 | 0.0003 | 0.0026 | 0.0003 | 0.626 |
| Tapetum_L | 0.0028 | 0.0004 | 0.0025 | 0.0003 | 0.078 |
